# Cyclin E modulates vulnerability to CDC7 kinase inhibition

**DOI:** 10.1038/s41389-026-00613-5

**Published:** 2026-04-24

**Authors:** Adam P. Dommer, Robert Kyne, Jianxin Wang, Thomas N. O’Connor, Amnon Koren, Erik S. Knudsen, Agnieszka K. Witkiewicz

**Affiliations:** 1 Department of Molecular and Cellular Biology, Roswell Park Comprehensive Cancer Center, Buffalo, NY USA; 2 Department of Pathology, Roswell Park Comprehensive Cancer Center, Buffalo, NY USA

**Keywords:** Breast cancer, DNA replication

## Abstract

*CCNE1* (cyclin E) is frequently amplified or overexpressed in triple-negative breast cancer (TNBC) as compared with luminal subtypes. Cyclin E is associated with chromosomal instability and poor outcome, and overexpression promotes replication stress (fork stalling) in S-phase through impaired MCM chromatin loading and deregulated replication origin firing. Thus, approaches leveraging cyclin E-induced replication stress could lead to the development of promising therapeutic strategies. Here, we studied the effects of cell division cycle 7 (CDC7) kinase inhibition in TNBC cells overexpressing cyclin E. Cyclin E overexpression enhanced sensitivity to CDC7 inhibition, reducing proliferation and colony-forming capacity. This was accompanied by delays in replication timing and cell accumulation with ≥4 N DNA content. Conversely, *CCNE1* knockdown rescued proliferation and colony outgrowth in the presence of CDC7 inhibition and reversed accumulation with ≥ 4N DNA content. CRISPR screening revealed cyclin-dependent kinase 8 (CDK8) as conferring resistance to CDC7 inhibition in a *CCNE1*-amplified cell line. Combined CDC7 and CDK8 inhibition significantly reduced proliferation and colony-forming ability, led to ≥4 N DNA content, and reduced tumor volume and mass in vivo. Together, this work identifies the enhanced vulnerability of cyclin E-overexpressing TNBC cells to CDC7 kinase inhibition and substantial synergy when combined with CDK8 inhibition.

## Introduction

Mitogenic stimuli impinge on cyclin D-CDK4/6, which in turn partially phosphorylates and inactivates the retinoblastoma (RB) tumor suppressor to enable cell cycle progression. Cyclin E/CDK2 then further phosphorylates RB, allowing E2F transcription factor expression of genes required for origin licensing and S-phase progression [1–3]. Deregulation of cyclin E1 through *CCNE1* amplification, *FBXW7* loss of function, or epigenetic alterations increases tumorigenesis and enhances resistance to CDK4/6 inhibition [3]. Cyclin E is also frequently overexpressed in triple-negative breast cancer (TNBC) compared with luminal subtypes [4–6]. Despite its oncogenic properties, however, cyclin E increases replication stress (replication fork stalling) in S-phase, which can be leveraged for therapeutic intervention.

Replication stress refers to the frequent stalling of replication forks due to exogenous or endogenous stressors resulting in DNA lesions or nucleotide depletion [7, 8]. Upon DNA polymerase stalling, helicases continue unwinding DNA, exposing single-stranded DNA. This is recognized and bound by RPA, ATR-interacting protein (ATRIP), and subsequently ATR, which engages in DNA repair and inhibition of mitotic entry through CHK1 and WEE1 activation [9–13]. Importantly, cyclin E/CDK2 enhances replication stress through a variety of different mechanisms. Aberrant cyclin E levels in late M/early G1 phase dampen minichromosome maintenance (MCM) protein loading onto chromatin, decreasing the abundance of dormant origins to be used during replication stress [14–18]. Additionally, deregulated origin firing mediated by cyclin E increases nucleotide consumption and DNA intermediates (e.g., replication/transcription collisions) [15, 16, 18–21]. This may result in competition for nucleotides and DNA damage repair machinery between active replication forks. Collectively, suboptimal maintenance of cyclin E/CDK2 activity increases replication stress in S-phase.

We hypothesized that cyclin E levels would directly influence dependency on cell division cycle 7 (CDC7) kinase for DNA replication. CDC7 is a serine/threonine kinase that binds its regulatory counterpart DBF4 or DBF4B and phosphorylates MCM2, resulting in CDC45/MCM/GINS (CMG) complex formation and bidirectional replication origin firing and fork progression [22–25]. CDC7 is also required for CHK1 recruitment during the DNA damage response [26–28]. Targeting the replication machinery via CDC7 inhibition has become a relevant therapeutic approach in solid and hematological cancers [29–31]. Importantly, aberrant cyclin E levels persisting into telophase limit chromatin loading of MCM-4 and MCM-7 subunits, leading to defects in S-phase progression [14]. Conceptually, CDC7 inhibition in cyclin E deregulated cells may interfere with origin firing in cells already experiencing limited amounts of chromatin-associated MCM, resulting in fewer active replication forks and enhanced dependency on these forks to travel greater distances for DNA replication, affecting the replication timing program. Lastly, we utilize CRISPR screening to identify genetic interactions involving CDC7 in a *CCNE1*-amplified cell line to extend CDC7 inhibitor combinatorial strategies beyond DNA-damaging chemotherapies; these findings may enhance the translational relevance of CDC7 inhibition by decreasing the lower effective dose required for response [31, 32].

## Results

### CDC7 inhibition is efficacious in *CCNE1*-amplified TNBC

We hypothesized that *CCNE1* amplification or overexpression would sensitize cells to CDC7 inhibition through the inherent replication stress mediated by cyclin E. Mining of the DepMap project revealed stronger correlations between *CDC7* vulnerability and *CCNE1* expression relative to previously published *PKMYT1* or *WEE1* vulnerabilities as a function of *CCNE1* expression in breast carcinomas **(**Fig. S1A–D**)** [4, 33]. Moreover, mining of The Cancer Genome Atlas (TCGA) revealed increased transcriptional levels of *CCNE1*, *CDC7* and *DBF4* in basal-type breast cancers compared to non-tumor adjacent tissue and luminal and HER-2-enriched subtypes **(**Fig. S1E**)**. Metabric data of TNBC patients further confirmed *CCNE1* copy number gain and gene amplification with increased *CCNE1* expression in a subset of individuals **(**Fig. S1F**)**, and that *CCNE1* and *CDC7* expression were positively correlated **(**Fig. S1G**)**. While high *CCNE1* and *CDC7* expression had no significant impact on duration of recurrence-free survival (RFS) or overall survival (OS) in TNBC patients, both RFS and OS were shorter in *CCNE1* and *CDC7-*high expressing individuals when assessed from all breast cancer patients regardless of subtype **(**Fig. S1H**)**.

**Fig. 1:**
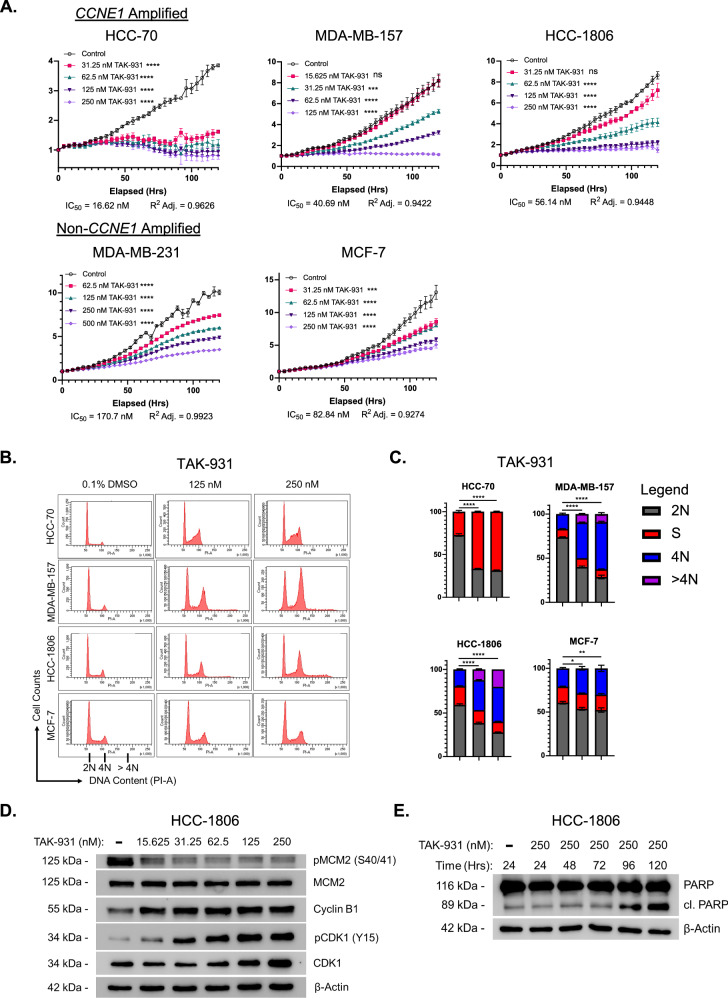
CDC7 inhibition is efficacious in *CCNE1*-amplified TNBC. **A** *CCNE1*-amplified or non-amplified breast cancer cells treated with serial concentrations of TAK-931 for five days and monitored with Cellcyte or Incucyte live cell proliferative software (*n* = 4; one-way ANOVA with Dunnett’s multiple comparisons test comparing each concentration to DMSO control using endpoint normalized GFP counts; error bars represent SEM). **B** Representative cell cycle profiling of the indicated cell lines following 48 hr treatment with TAK-931. **C** Quantification of cell cycle state from indicated cell lines treated with 48 hr TAK-931 (*n* = 3; one-way ANOVA with Dunnett’s multiple comparisons test comparing each concentration to DMSO control with significance determined for ≥ 4 N DNA (for HCC-70, significance determined for >2 N DNA); error bars represent SD). **D** Representative western blot of HCC-1806 cells for pMCM2 (S40/41) and mitotic inhibition following 48 hr TAK-931 treatment. **E** Representative western blot of HCC-1806 cells for cleaved PARP following time-dependent exposure to 250 nM TAK-931.

For this study, we utilized the ATP-competitive CDC7 inhibitors TAK-931 and XL-413. Live cell proliferative analysis demonstrated pronounced antiproliferative effect in all *CCNE1*-amplified TNBC cells treated with CDC7 inhibitor as compared with non-amplified cells. **(**Fig. 1A, S1I**)**. Following five-day exposure to CDC7 inhibition, cells appeared with pyknotic (indicative of apoptosis) or grossly enlarged nuclei, which was less pronounced in non-*CCNE-*amplified cells **(**Fig. S1J**)**. Importantly, CDC7 inhibition caused accumulation with ≥ 4 N DNA content that was less prominent in non-*CCNE1*-amplified cells **(**Fig. 1B, C, Fig. S1K, L**)**. Abrogating CDC7 function led to diminished MCM2 phosphorylation, cyclin B1 accumulation and CDK1 phosphorylation (Y15), corroborating the cell accumulation with ≥4 N DNA content **(**Fig. 1D, S1M**)**. Moreover, CDC7 inhibition increased cleaved PARP over time, consistent with the pyknotic morphology observed at the endpoint of live cell proliferative analysis **(**Fig. 1E, S1N**)**. To substantiate the additional role of CDC7 in intra-S phase signaling, the DNA damage response was induced using aphidicolin to generate single-stranded DNA. Treatment with aphidicolin led to phosphorylated CHK1 (S317, 345), which was reversed upon co-treatment with TAK-931, thus reinforcing the role of CDC7 in CHK1 activation **(**Fig. S1O**)**. Collectively, all tested *CCNE1*-amplified TNBC cells responded to CDC7 inhibition with halted proliferation, ≥4 N DNA content, and signs of apoptosis.

### *CCNE1* knockdown reverses sensitivity to CDC7 inhibition

**Fig. 2:**
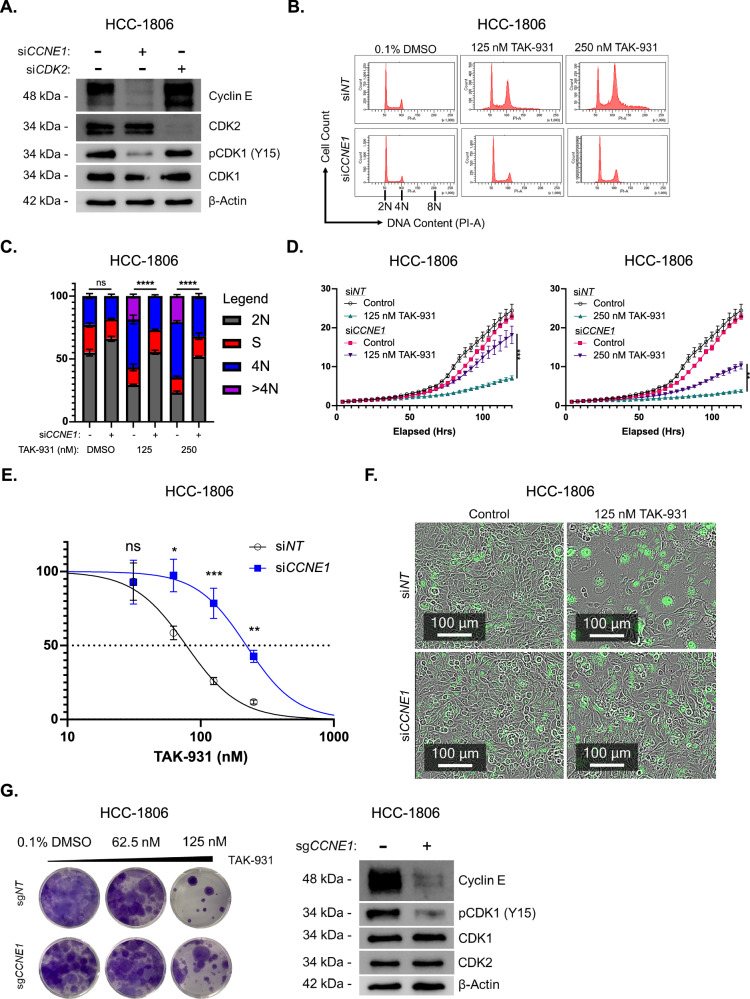
*CCNE1* knockdown reverses sensitivity to CDC7 inhibition. **A** Representative western blot of HCC-1806 cells for cyclin E, CDK2, and pCDK1 (Y15) following *CCNE1* or *CDK2* knockdown. **B** Representative cell cycle profiling of HCC-1806 cells following *CCNE1* knockdown and 48 hr treatment with indicated concentrations of TAK-931. **C** Quantification of cell cycle state from HCC-1806 cells following *CCNE1* knockdown and 48 hr treatment with indicated concentrations of TAK-931 (n = 3; two-way ANOVA with Tukey’s multiple comparisons test accounting for gene and drug effect with significance determined for ≥ 4 N DNA; error bars represent SD). **D** HCC-1806 cells following *CCNE1* knockdown and treatment with 125 nM (left) or 250 nM (right) TAK-931 for five days and monitored with Cellcyte or Incucyte live cell proliferative software (*n* = 4; two-way ANOVA with Tukey’s multiple comparisons test accounting for gene and drug effect using endpoint normalized GFP counts; error bars represent SEM). **E** IC_50_ curves derived from live cell proliferative analyses as described in (**D**) (*n* = 4; two-way ANOVA with Tukey’s multiple comparisons test accounting for gene and drug effect using endpoint normalized GFP counts; error bars represent SEM). **F** Representative images captured at the end of five-day live cell proliferative analyses from HCC-1806 cells following *CCNE1* knockdown and treatment with 125 nM TAK-931. **G** Representative colony outgrowth assay from HCC-1806 cells with *CCNE1* CRISPR knockout or non-targeting control and treatment with indicated concentrations of TAK-931 for three cycles, with treatment replenishment every five days (left) and western blot of HCC-1806 CRISPR *CCNE1* isogenic pair for cyclin E, CDK2, and pCDK1 (Y15) (right).

As CDC7 kinase is required for replication origin firing and CHK1 signaling, we hypothesized that cyclin E-overexpressing cells would have enhanced dependency on CDC7 for proliferation. Intriguingly, using the HCC-1806 TNBC cells that can proliferate independently of cyclin E/CDK2, we found that only *CCNE1* knockdown dampened the G2/M checkpoint response mediated through phosphorylated CDK1 (Y15); however, this may be consistent with CDK2-independent roles of cyclin E in MCM loading and cell transformation **(**Fig. 2A**)** [34, 35]. Importantly, *CCNE1* knockdown significantly reversed cell cycle accumulation with ≥ 4 N DNA content in the presence of CDC7 inhibitor **(**Figs. 2B, C, S2A–C**)**. This was accompanied by significant desensitization of cells to the antiproliferative effects of CDC7 inhibition **(**Figs. 2D–GS2D–H**)**. Interestingly, CDC7 inhibition led to diminished cyclin E levels, possibly as a collateral effect of the ≥ 4 N DNA arrest. **(**Fig. S2I**)**. These findings directly implicate cyclin E as a modulator of response to CDC7 inhibition.

### Cyclin E overexpression enhances sensitivity to CDC7 inhibition

**Fig. 3:**
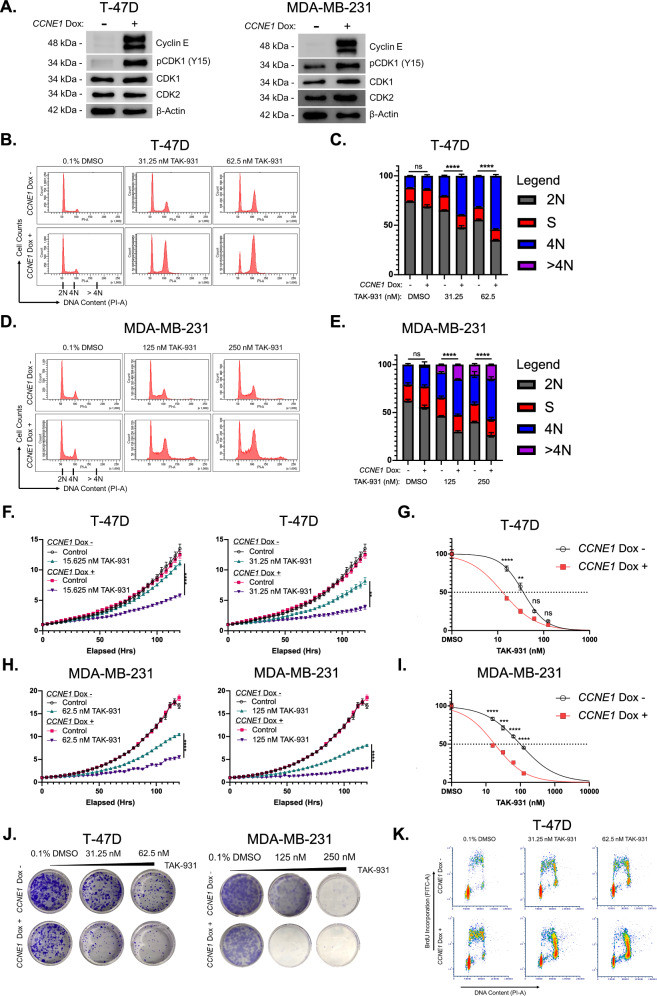
Cyclin E overexpression enhances sensitivity to CDC7 inhibition. **A** Representative western blot of T-47D (left) and MDA-MB-231 (right) cells for cyclin E, CDK2, and pCDK1 (Y15) following *CCNE1* induction. **B** Representative cell cycle profiling of T-47D cells following *CCNE1* induction and 48 hr treatment with indicated concentrations of TAK-931. **C** Quantification of cell cycle state from T-47D cells following *CCNE1* induction and 48 hr treatment with indicated concentrations of TAK-931 (*n* = 3; two-way ANOVA with Tukey’s multiple comparisons test accounting for gene and drug effect with significance determined for ≥ 4 N DNA; error bars represent SD). **D** Representative cell cycle profiling of MDA-MB-231 cells following *CCNE1* induction and 48 hr treatment with indicated concentrations of TAK-931. **E** Quantification of cell cycle state from MDA-MB-231 cells following *CCNE1* induction and 48 hr treatment with indicated concentrations of TAK-931 (*n* = 3; two-way ANOVA with Tukey’s multiple comparisons test accounting for gene and drug effect with significance determined for ≥ 4 N DNA; error bars represent SD). **F** T-47D cells following *CCNE1* induction and treatment with 15.625 nM (left) or 31.25 nM (right) TAK-931 for five days and monitored with Cellcyte or Incucyte live cell proliferative software (*n* = 4; two-way ANOVA with Tukey’s multiple comparisons test accounting for gene and drug effect using endpoint normalized GFP counts; error bars represent SEM). **G** IC_50_ curves derived from live cell proliferative analyses as described in (**F**) (*n* = 4; two-way ANOVA with Tukey’s multiple comparisons test accounting for gene and drug effect using endpoint normalized GFP counts; error bars represent SEM). **H**, MDA-MB-231 cells following *CCNE1* induction and treatment with 62.5 nM (left) or 125 nM (right) TAK-931 for five days and monitored with Cellcyte or Incucyte live cell proliferative software (*n* = 4; two-way ANOVA with Tukey’s multiple comparisons test accounting for gene and drug effect using endpoint normalized GFP counts; error bars represent SEM). **I** IC_50_ curves derived from live cell proliferative analyses as described in (**H**) (*n* = 4; two-way ANOVA with Tukey’s multiple comparisons test accounting for gene and drug effect using endpoint normalized GFP counts; error bars represent SEM). **J** Representative colony outgrowth assays from T-47D (left) and MDA-MB-231 (right) cells following *CCNE1* induction and treatment with indicated concentrations of TAK-931 for three cycles, with doxycycline and treatment replenishment every five days. **K** Representative BrdU incorporation analysis of T-47D cells following *CCNE1* induction and 48 hr treatment with indicated concentrations of TAK-931.

To directly assess the impact of cyclin E overexpression on sensitivity to CDC7 inhibition, functional studies were conducted with T-47D (ER+/HER2-) and MDA-MB-231 (TNBC) cell lines placed under the control of doxycycline (Dox)-inducible *CCNE1*. Two-day exposure to Dox led to cyclin E overexpression and phosphorylated CDK1 (Y15), indicating the effects of cyclin E alone on G2/M checkpoint signaling **(**Fig. 3A**)**. Notably, cyclin E and phospho-CDK1 (Y15) levels post-Dox induction were comparable to baseline levels in the *CCNE1*-amplified cell line HCC-1806 **(**Fig. S3A**)**. Cells overexpressing cyclin E were more vulnerable to CDC7 inhibition, evidenced by significantly increased accumulation with ≥ 4 N DNA content **(**Fig. 3B–E, S3B–G**)**. Moreover, cyclin E overexpression significantly reduced proliferation and colony-forming ability in cells treated with CDC7 inhibitor **(**Fig. 3F–J, S3H–M**)**. BrdU incorporation assays revealed enhanced S-phase cell accumulation upon cyclin E overexpression alone, and CDC7 inhibition increased cell accumulation with ≥ 4 N DNA content **(**Fig. 3K**)**. Cyclin E expression was again found to be reversed upon CDC7 inhibition **(**Fig. S3N**)**. Together, cyclin E overexpression increased G2/M checkpoint signaling and significantly enhanced sensitivity to CDC7 inhibition.

### CDC7 inhibition associates with delayed replication timing in Cyclin E deregulated cells

**Fig. 4:**
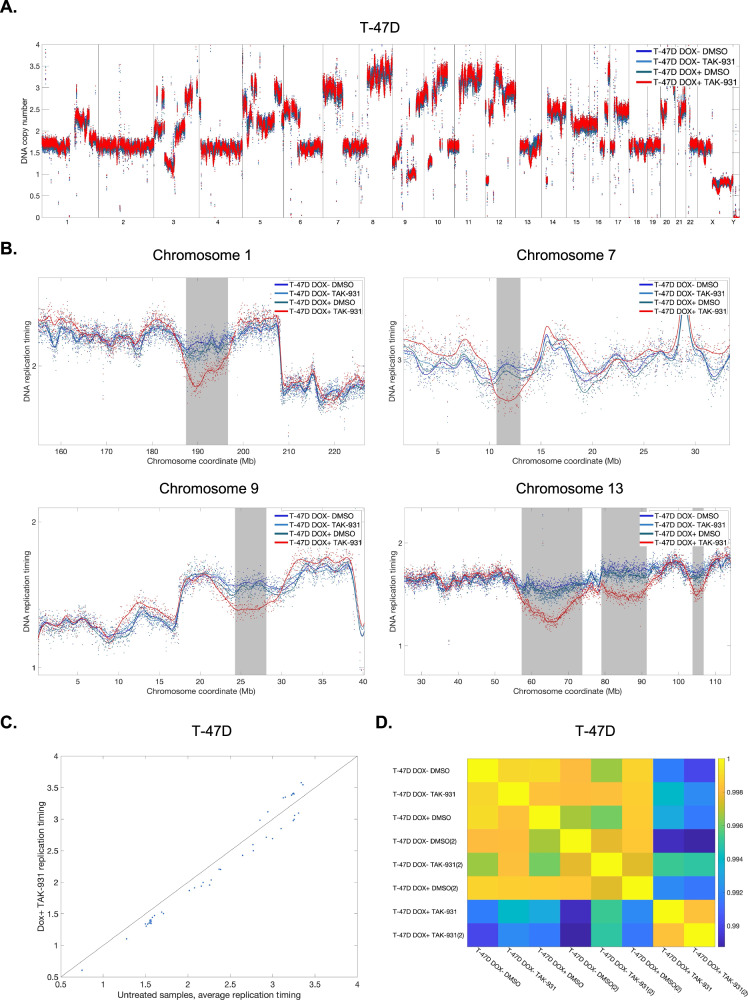
CDC7 inhibition associates with delayed replication timing in Cyclin E deregulated cells. **A** Representative overlay of genome-wide replication timing profiles from T-47D cells following *CCNE1* induction and 48 hr treatment with 62.5 nM TAK-931. **B** Representative individual chromosome replication timing profiles from T-47D cells following *CCNE1* induction and 48 hr treatment with 62.5 nM TAK-931. **C** Representative correlation plot depicting delayed replication timing of T-47D cells following *CCNE1* induction and 48 hr treatment with 62.5 nM TAK-931. **D** Representative heatmap depicting correlations in replication timing as discussed in (**A**–**C)**.

We next investigated whether CDC7 inhibition in cyclin E overexpressing cells perturbs DNA replication, resulting in the observed accumulation with ≥ 4 N DNA content. Conceptually, CDC7 perturbation in cells with improper MCM loading may influence replication fork activity and subsequent replication timing. To profile DNA replication timing, we sequenced the genomes of proliferating cells and analyzed fluctuations in DNA copy number, with lower copy number inferring delayed timing [36]. We systemically identified regions with altered replication timing in samples with varied cyclin E levels and CDC7 activity **(**Figs. 4A, S4A**)**. Individual overexpression of cyclin E or CDC7 inhibition had little effect on replication dynamics **(**Fig. 4B, S4B, C**)**. However, cyclin E overexpression in conjunction with CDC7 inhibition led to delays of replication timing indicative of challenges in completing DNA replication—many of these regions spanned megabases of DNA, with some larger than 10 megabase pairs **(**Figs. 4B, S4B, C**)**. Instances of advanced replication timing in cyclin E-overexpressing cells treated with CDC7 inhibitor were less prominent than delays **(**Figs. 4C, S4C**)**. The strongest inverse correlations in replication timing were observed between *CCNE1* non-induced, DMSO-treated cells and *CCNE1*-induced, CDC7 inhibitor-treated cells **(**Fig. 4D**)**. Collectively, CDC7 inhibition delays replication timing in cyclin E deregulated cells.

### Cyclin E impacts MCM chromatin reloading in cells with 4 N DNA content

**Fig. 5:**
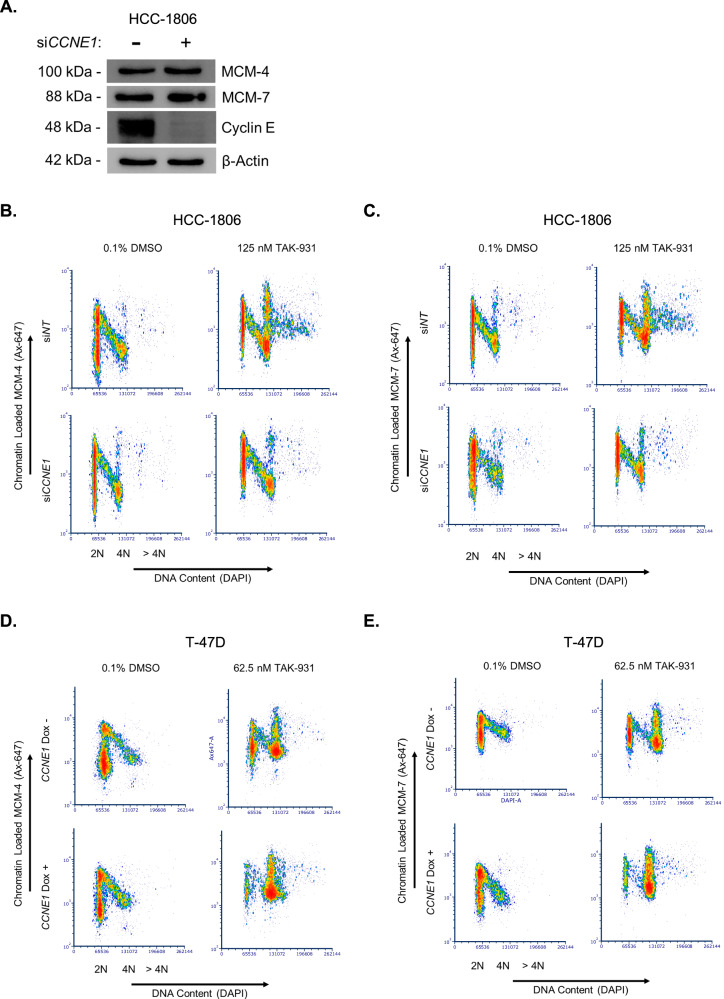
Cyclin E impacts MCM chromatin reloading in cells with 4 N DNA content. **A** Representative western blot of HCC-1806 cells for total MCM-4, 7, and cyclin E following *CCNE1* knockdown. **B** Representative flow cytometric MCM-4 chromatin binding assay from HCC-1806 cells following *CCNE1* knockdown and 48 hr treatment with 125 nM TAK-931. **C** Representative flow cytometric MCM-7 chromatin binding assay from HCC-1806 cells following *CCNE1* knockdown and 48 hr treatment with 125 nM TAK-931. **D** Representative flow cytometric MCM-4 chromatin binding assay from T-47D cells following *CCNE1* induction and 48 hr treatment with 62.5 nM TAK-931. **E** Representative flow cytometric MCM-7 chromatin binding assay from T-47D cells following *CCNE1* induction and 48 hr treatment with 62.5 nM TAK-931.

To interrogate the chromatin loading dynamics of MCM subunits whose loading is altered in cyclin E deregulated cells, MCM-4/7 chromatin binding analysis was performed using flow cytometry. As expected, *CCNE1* knockdown alone did not alter total MCM levels, consistent with its effect in perturbing MCM chromatin association rather than total MCM pools **(**Fig. 5A**)**. Intriguingly, cells arresting with ≥ 4 N DNA content following CDC7 inhibition exhibited increased chromatin loading for both MCM-4 and MCM-7 subunits **(**Fig. 5B–E**)**. *CCNE1* knockdown reversed the number of cells with chromatin-bound MCM in cells with ≥ 4 N DNA following CDC7 inhibition **(**Fig. 5B, C**)**. On the other hand, CDC7 inhibition mitigated MCM origin loading in G1 and exacerbated reloading in cells with ≥ 4 N DNA content following cyclin E overexpression **(**Fig. 5D, E**)**. Collectively, increased cyclin E levels directly enhance the reloading of MCM helicases in cells with ≥ 4 N DNA content following CDC7 inhibition.

### CDK8 inhibition synergizes with CDC7 inhibition in *CCNE1*-amplified cells

**Fig. 6:**
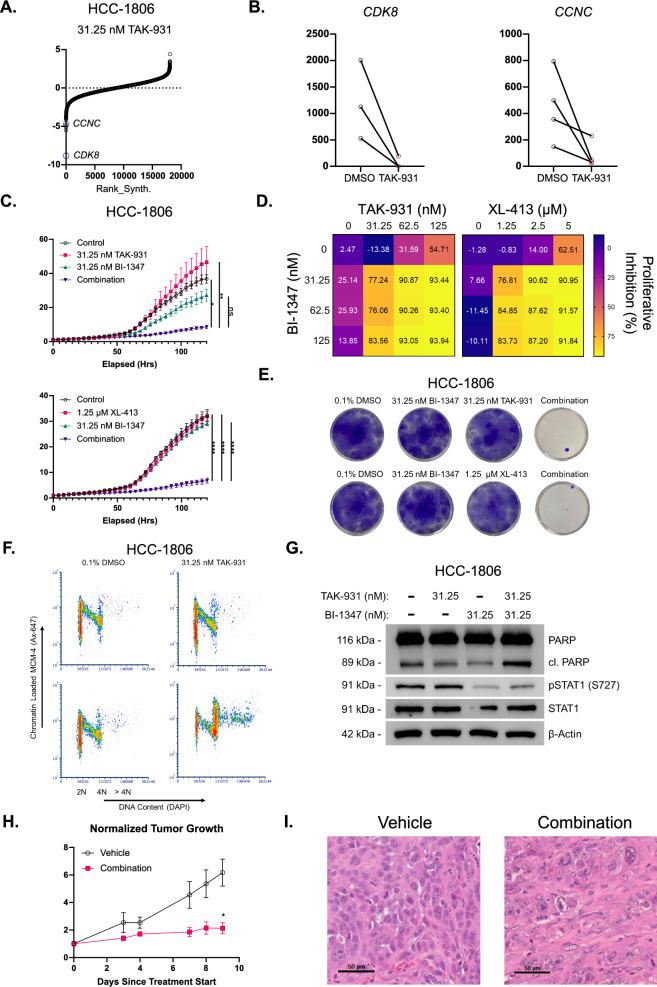
CDK8 inhibition synergizes with CDC7 inhibition in *CCNE1*-amplified cells. **A** S-curve depicting normalized z-scores for transcript guides following CRISPR screening in HCC-1806 cells treated with 31.25 nM TAK-931. **B** Individual guide dropout rates for *CDK8* and *CCNC* from HCC-1806 cells following CRISPR screening. **C** HCC-1806 cells following treatment with combined TAK-931 and BI-1347 (top) or XL-413 and BI-1347 (bottom) for five days and monitored with Cellcyte or Incucyte live cell proliferative software (n = 4; one-way ANOVA with Tukey’s multiple comparisons test comparing each treatment condition using endpoint normalized GFP counts; error bars represent SEM). **D** Heatmap depicting endpoint proliferative inhibition from live cell proliferative analyses as described in (**C**). **E** Representative colony outgrowth assays from HCC-1806 cells following treatment with combined TAK-931 and BI-1347 (top) or XL-413 and BI-1347 (bottom) for three cycles, with treatment replenishment every five days. **F** Representative flow cytometric MCM-4 chromatin binding assay from HCC-1806 cells following treatment with TAK-931, BI-1347, or combination for 48 h. **G** Representative western blot of HCC-1806 cells for cleaved PARP and pSTAT1 (S727) following treatment with TAK-931, BI-1347, or combination for 120 h. **H** Normalized tumor volume since treatment initiation from in vivo HCC-1806 xenograft models orally administered combined TAK-931 and BI-1347 (*n* = 4 per vehicle group and 5 per combination group; unpaired t-test with Welch’s correction (two-tailed); error bars represent SEM). **I** Representative H&E staining of tumors excised at endpoint from in vivo study as described in (**H**).

To limit the effective dose of CDC7 inhibitor required for response beyond combination strategies involving DNA-damaging chemotherapies, we utilized CRISPR screening to identify proteins conferring resistance to CDC7 inhibition in a *CCNE1*-amplified cell line. HCC-1806 cells were infected with a sgRNA CRISPR library, passaged in the presence of low concentrations of TAK-931, and analyzed for differential guide dropout rates [37]. Notably, *CCNC* and *CDK8* guides had significantly enhanced dropout rates in cells treated with CDC7 inhibitor, whereas the *CDK8* paralog *CDK19* did not display differential dropout frequencies **(**Fig. 6A, B, S5A**)**. Cyclin C/CDK8 forms the kinase module of the mediator complex required for transcriptional control; however, we did not observe enhanced dropout of the regulatory mediator members, suggesting a mediator-independent role of cyclin C/CDK8 in protecting cells from CDC7 inhibitory effects as well **(**Fig. S5A**)** [38, 39]. Live cell proliferation analysis confirmed synergy between both CDC7 inhibitors and either *CDK8* knockdown or the CDK8-selective inhibitor BI-1347 **(**Fig. 6C, D, S5B, C**)**. Cells treated with combined CDC7 and CDK8 inhibitor also displayed significantly blunted colony outgrowth ability **(**Fig. 6E**)**. While univariate cell cycle profiling revealed enrichment of cells with ≥4 N DNA content, chromatin binding assays demonstrated MCM-4 reloading in these cell populations **(**Figs. 6F, S5D**)**. BI-1347 selectivity was confirmed through reduced STAT1 phosphorylation (pSTAT1 S727), and combined CDC7 and CDK8 inhibition led to increased cleaved PARP, indicating apoptosis **(**Figs. 6G, S5E**)** [40]. These findings indicate that CDK8 protects cells from the effects of CDC7 inhibition.

To validate CDC7/CDK8 interaction in vivo, HCC-1806 cells were implanted into the mammary fat pad of female NOD scid gamma (NSG) mice, followed by oral administration of TAK-931 and BI-1347 corresponding to the treatment regimen outlined in Fig. S5F. Mice administered both TAK-931 and BI-1347 had significantly reduced tumor growth and endpoint tumor mass compared with vehicle-treated mice **(**Figs. 6H, S5G**)**. H&E staining of tumors excised at endpoint revealed enlarged nuclear morphology as observed in vitro in cells accumulating with ≥ 4 N DNA content and MCM-4 reloading **(**Fig. 6I**)**. Together, combined inhibition of CDC7 and CDK8 is a promising approach for *CCNE1*-amplified disease control.

## Discussion

*CCNE1* is often amplified or overexpressed in TNBC and is associated with chromosomal instability and poor outcome [4–6]. Importantly, cyclin E induction of replication stress in S-phase represents a promising vulnerability for therapeutic intervention. While prior studies with CDK2 selective inhibitors have been limited due to off-target CDK1 inhibition, recent developments in leveraging cyclin E oncogenic stress through G2/M checkpoint kinase inhibition reveal enhanced DNA damage and mitotic catastrophe that is abrogated following *CCNE1* ablation [4, 5, 15, 16, 18, 21, 33, 41, 42]. Here, we find that cells harboring *CCNE1* amplification have heightened sensitivity to inhibition of CDC7 kinase – a serine/threonine kinase essential for replication origin firing and intra-S phase signaling. While other determinants of response to CDC7 inhibition have been demonstrated (e.g., *TP53*, *RIF1*), we find that modulation of cyclin E levels directly influences response to CDC7 inhibition [43–45]. Intriguingly, we find that cyclin E modulation directly impacts CDK1 phosphorylation (inactivation) independently of CDK2. Moreover, *CCNE1* knockdown mitigates the response to the antiproliferative effects of CDC7 inhibition. Conversely, cyclin E1 overexpression enhances sensitivity to CDC7 kinase inhibition, evidenced by increased antiproliferative effect and cell accumulation with ≥ 4 N DNA content. It is possible, however, that the window for therapeutic intervention utilizing CDC7 inhibitors in *CCNE1*-amplified cancers is greater than approaches utilizing G2/M inhibitors such as ATR, CHK1, and WEE1 inhibitors. While checkpoint kinase function is imperative in cells with enhanced replication stress, CDC7/Dbf4 is critical in S-phase initiation in most cells. CDC7 is also required for homologous recombination, MRE-11-mediated fork processing, and regulation of EXO1 nascent strand degradation following replication stress [32, 46, 47]. Moreover, this study utilizes TNBC (HCC-70, HCC-1806, MDA-MB-157, MDA-MB-231) and ER+/HER2- (T-47D, MCF-7 (*TP53* WT)) breast cancer cell lines with differential cyclin E levels and dependencies and *TP53* mutational signatures, the latter of which being imperative in the licensing checkpoint and mediating resistance to CDC7 inhibitors through reversible cell cycle arrest [22, 43, 48, 49]. Importantly, while *CCNE1* induction alone in T-47D cells does not define transition from ER+/HER2- to basal-like (TNBC) breast cancer, it induces TNBC-like properties associated with enhanced resistance to CDK4/6 inhibition and elevated replication stress and genome instability [4, 5, 19, 20, 50]. Utilization of the *CCNE1*-amplified, but non-dependent, HCC-1806 cell line permits modulation of cyclin E-induced replication stress without affecting baseline fitness.

Inappropriate cyclin E levels in late M/early G1 phase prevent the proper distribution of MCM helicases onto chromatin prior to S-phase [14–17, 48, 51]. In this scenario, it is possible that CDC7 inhibition further exacerbates replication stress and places heightened dependency on active replication forks to travel greater distances for DNA replication, impacting replication timing at vulnerable genomic regions. While CDC7 inhibition may rescue individual fork progression rates in cyclin E deregulated cells, our data suggest that the combination is ultimately detrimental to DNA replication dynamics and proliferation [20, 52]. We find that CDC7 inhibition in cyclin E-overexpressing cells delays replication timing of megabase regions of DNA, although it remains to be investigated which genomic regions are being impacted, whether they comprise susceptible regions (e.g., common fragile sites), and additional phenotypic consequences. Strikingly, we find that cells accumulating with ≥4 N DNA following CDC7 inhibition reload MCM subunits onto chromatin, possibly indicating preparation for an additional S-phase in the absence of mitosis. This effect is ameliorated in cells lacking cyclin E. We find this approach to be distinct from G2/M inhibition in *CCNE1*-amplified cells. Rather than forcing cells with DNA damage into premature mitosis and catastrophe, we observe a further delay in genome duplication at distinct genomic loci, resulting in perturbed proliferation, attempted re-replication, and cell death. Collectively, these data imply that heightened sensitivity to CDC7 inhibition in cyclin E-deregulated cells is accommodated by perturbed replication dynamics and increased MCM chromatin reloading in cells with ≥ 4 N DNA.

CDC7 inhibitors have demonstrated limited preclinical efficacy and significant side effects that include cytopenia [31]. Drug screens have revealed synergy between CDC7 inhibitors and DNA-damaging chemotherapies [32]. Here, we highlight an opportunity to extend CDC7 inhibitor utility through its synergetic use with CDK8 inhibitors pertaining to a mediator-independent function of CDK8. Future studies would benefit from building upon these findings by identifying other indications for which CDC7 kinase and CDK8 inhibition could prove beneficial and elucidating ways to minimize toxicity and improve therapeutic translation. Notably, it has been demonstrated that inhibition of the CDK8/19 mediator kinase module overcomes transcriptional rewiring following PI3K/AKT/mTOR inhibition in TNBC to reverse acquired resistance initially driven by compensatory transcriptional rewiring following treatment [53, 54]. It would be interesting to determine whether CDC7 inhibition imparts distinct transcriptional programs that are reversed following CDK8 inhibition. Lastly, the successful development of CDK2-specific inhibitors has revealed prominent cytostatic efficacy in *CCNE1*-amplified or dependent TNBC and gynecological models, revitalizing cytostatic efforts in addition to CDK1 activation [55–59]. Importantly, innate resistance to CDK4/6 inhibitors is often accompanied by cyclin E overexpression, as is acquired resistance via *CCNE1* amplification or non-canonical cyclin D/CDK2 activity [50, 60–64]. It is possible that cells utilizing cyclin E to overcome CDK4/6 inhibitor resistance experience enhanced replication stress that conveys hypersensitization to CDC7 inhibition as well. It would be advantageous to examine whether adaptive resistance to CDK4/6 inhibitors mediated by cyclin E portends CDC7 inhibitor sensitivity.

## Materials and Methods

### Compounds

TAK-931 (HY-100888, 2 mg), XL-413 Monohydrochloride (HY-15260A, 5 mg), and BI-1347 (HY-120350, 5 mg) were obtained from MedChemExpress. For in vivo studies, TAK-931 (CT-TAK931) was obtained from ChemieTek, and BI-1347 (HY-120350) was obtained from MedChemExpress.

### Cell culture

Cell lines were obtained from ATCC or Sigma-Aldrich. Cell line identity was validated by short tandem repeat testing and mycoplasma testing was performed regularly. HCC-1806 (CRL-2335, RRID:CVCL_1258), HCC-70 (CRL-2315, RRID:CVCL_1270) and T-47D (HTB-133, RRID:CVCL_0553) cells were cultured in RPMI-1640 medium (Corning, 10 040-CV) supplemented with 10% fetal bovine serum (FBS, Gibco, A52567-01) and 1% antibiotic-antimycotic (Gibco, 15240-062). MDA-MB-157 (HTB-24, RRID:CVCL_0618), MDA-MB-231 (HTB-26, RRID:CVCL_0062), and MCF-7 (HTB-22, RRID:CVCL_0031) cells were cultured in DMEM medium (Corning, 10-013-CV) supplemented with 10% FBS and 1% antibiotic antimycotic. Dulbecco’s phosphate-buffered saline (DPBS, Corning, 21-031-CV) was used to remove excess media prior to trypsinization. Cells were detached from culturing plates with 0.25% trypsin (Corning, 25-053-Cl).

### External datasets and plotting

Three external datasets were used in this study. Gene effect and expression (25Q2 release) were downloaded from DepMap portal and used to generate expression and vulnerability plots for select cell cycle genes. Predictability and expression data were downloaded directly from the DepMap website (depmap.org). Breast cancer data from The Cancer Genome Atlas (Breast Invasive Carcinoma (TCGA, PanCancer Atlas) was downloaded from cBioPortal (www.cbioportal.org). Metabric datasets for gene expression, copy number, and clinical outcomes were downloaded from cBioPortal (https://www.cbioportal.org/study/summary?id=brca_metabric). TNBC patients were selected by setting ER_STATUS == ‘Negative’, HER2_STATUS == ‘Negative’, PR_STATUS == ‘Negative’ in an R script using “data_clinical_sample.txt” as input. The boxplot of *CCNE1* expression and mutational status was generated using the ggpubr (v0.6.2) R package. Survival data was filtered by selecting patients with OS_MONTHS < 220. Kaplan-Meier plots were made with a custom R script utilizing the ggplot2 (v4.0.0), survival (v3.8-3) and survminer (v0.5.1) R packages. Each group in the Kaplan-Meier plots was stratified by median gene expression values. This resulted in three groups (CCNE1.CDC7.exp = =High: patients with expression values of both genes above the median of each gene; CCNE1.CDC7.exp == Low, patients with expression values of both genes below the median of each gene; Other, patients with only one gene with an expression value above the median).

### Western blotting

Whole cell extracts were obtained by lysing cells in RIPA buffer (Santa Cruz Biotechnology, SC 24948 A) supplemented at 1:100 with phenylmethanesulfonyl fluoride (PMSF, Sigma) and Halt Protease Inhibitor Cocktail (ThermoFisher). Membranes were blocked in 5% milk (ChemCruz, sc-2325) prepared in tris-buffered saline tween (TBST) for one hour at room temperature (RT) while gently rocking. Primary antibodies were used at 1:1500 dilution in 5% bovine serum albumin (BSA)-supplemented TBST or 5% milk-supplemented TBST unless noted otherwise. Antibodies were obtained from the following vendors: Ph.RB (S807/811) (Cell Signaling Technology Cat# 8516, RRID:AB_11178658), CDK2 (Cell Signaling Technology Cat# 2546, RRID:AB_2276129), Ph.CDK1 (Y15) (Cell Signaling Technology Cat# 9111, RRID:AB_331460), CDK1 (Cell Signaling Technology Cat# 77055, RRID:AB_2716331), Cyclin B1 (Cell Signaling Technology Cat# 12231, RRID:AB_2783553), Cleaved PARP (Cell Signaling Technology Cat# 9542, RRID:AB_2160739), Ph.STAT1 (S727) (Cell Signaling Technology Cat# 8826, RRID:AB_2773718), STAT1 (Cell Signaling Technology Cat# 9172, RRID:AB_2198300), Ph.CHK1 (S317) (Cell Signaling Technology Cat# 2344, RRID:AB_331488), Ph.CHK1 (S345) (Cell Signaling Technology Cat# 2348, RRID:AB_331212), CHK1 (Cell Signaling Technology Cat# 2360, RRID:AB_2080320), CDK1 (Santa Cruz Biotechnology Cat# sc-54, RRID:AB_627224) (1:500), MCM-7 (Santa Cruz Biotechnology Cat# sc-9966, RRID:AB_627235) (1:500), Ph.MCM2 (S40/41) (Abcam Cat# ab70371, RRID:AB_2141957), Cyclin E (Bethyl Cat# A301-566A, RRID:AB_1039994), MCM-4 (Thermo Fisher Scientific Cat# MA5-52516, RRID:AB_3248993), β-actin (R and D Systems Cat# MAB8929, RRID:AB_3076436). Secondary antibodies were used at 1:2000 dilution in 5% milk-supplemented TBST for one hour at RT while gently rocking and obtained from: Anti-rabbit (Thermo Fisher Scientific Cat# A27036, RRID:AB_2536099), Anti-mouse (Santa Cruz Biotechnology Cat# sc-516102, RRID:AB_2687626). West Femto SuperStrength ECL (Thermo Scientific, PI34096) was used at a 1:1 ratio per reagent and applied to membranes directly before imaging on a BIO-RAD ChemiDoc MP Imaging System.

### Live-cell proliferative analysis

Cells were stably transfected with pLenti0.3UbCGWH2BC1-PatGFP to express H2B-GFP, and GFP-positive cells were selected using a BD FACSAria II cell sorter. Cells were seeded between 1500 and 5000 cells per well into clear-bottom 96-well plates (Corning, 3603). The following day, cells were treated with the corresponding drug and subjected to live-cell imaging using CellCyte or Incucyte for five days. Average GFP counts per field of view were taken for each well, and each time point was normalized to initial counts. Plots were generated as normalized GFP counts as a function of time. For knockdown studies, cells were reverse-transfected the day of seeding and treated the following day for five days of monitoring. For cyclin E overexpression studies, cells were induced with 2 μg/mL doxycycline the day of seeding and treated the following day for five days of monitoring.

### Knockdown studies

Cells were reverse-transfected with siRNA using Dharmacon ON-TARGET Smart Pools comprising several guides per transcript: Non-targeting (*NT*) control (D—001810- 10-05), *CDK2* (L-003236-00-0005), *CCNE1* (L-003213-00- 0005), and *CDK8* (L-003236-00-0005). Transfection was performed using reduced serum Opti-MEM (Gibco, 11058-021) and RNAiMAX lipofectamine reagent (Invitrogen, 13778-150). Cells were treated 24 hours post-transfection. Knockdown efficiency was confirmed by immunoblotting for the target protein.

### Overexpression studies

T-47D and MDA-MB-231 cells were transfected with pLentihygro4TRE3GV5-DEST-CCNE1 and selected with blasticidin. Doxycycline was added for cyclin E overexpression, which was confirmed using western blotting. Cells were induced with 2 μg/mL doxycycline and treated 24 hours post-induction. Overexpression efficiency was confirmed by immunoblotting for the target protein.

### Cell cycle profiling

Cells were seeded overnight into six-well plates and treated the following day with the drug at the indicated doses. Cells were trypsinized, and pellets rinsed once in PBS followed by fixation and permeabilization with 70% ethanol in PBS with vortexing. Samples were then stored at −20 °C overnight. The following day, samples were again rinsed with PBS, then aliquoted to FACS tubes, and RNAse A and PI were added to the samples at a final concentration of 200 μg/mL and 40 μg/mL, respectively. Samples were subsequently analyzed using an LSR Fortessa cytometer. 10,000 events were recorded per sample.

### Bromodeoxyuridine incorporation analysis

Cells were seeded overnight into six-well plates and treated the following day with the corresponding drug at the indicated dose for 48 hours, then pulse-labeled with BrdU (1:1000, Millipore Sigma #11669915001) for two hours. Cells were then harvested, rinsed in PBS, and fixed and permeabilized in 70% EtOH/PBS while vortexing and placed at −20 °C overnight. DNA was denatured with 2 N HCl/0.5% Triton X-100 for 30 minutes at RT. 0.1 M sodium tetraborate, pH 8.5, was added for 2 minutes at RT. Cell washes were conducted using 1% BSA in TBST. Anti-BrdU FITC (BD Bioscience, #556028) was prepared in 0.5% Tween 20/1% BSA/TBST and added to each sample for 1 hour at RT. Cells were then pelleted, rinsed once with 1% BSA/TBST, and aliquoted to FACS tubes. RNase A and PI were added to the samples at a final concentration of 200 μg/mL and 40 μg/mL, respectively. Samples were subsequently analyzed using an LSR Fortessa cytometer. 10,000 events were recorded per sample.

### Colony outgrowth assays

Cells were seeded at 1000 cells per well into six-well plates and adhered overnight, either with or without doxycycline for cyclin E overexpression studies. The following day, cells were treated with the corresponding drug with treatment medium replenished every five days (with or without 2 μg/mL doxycycline for cyclin E overexpression studies) for a total of three weeks. Upon endpoint, cells were fixed in 100% methanol at -20 °C for 10-15 minutes. Methanol was removed, and cells were stained in 0.5% crystal violet solution prepared in methanol and ddH_2_O for 15 minutes at RT. Crystal violet solution was removed 2-3 times with ddH_2_O rinses, and cells were inverted to dry overnight. Plates were imaged using a BIO-RAD ChemiDoc MP Imaging System. Colonies were quantified using the “ColonyArea” ImageJ plugin [65].

### Cytoskeletal buffer recipe

The cytoskeletal (CSK) buffer consists of 10 mM HEPES (pH 7.4), 150 mM NaCl, 300 mM sucrose, and 3 mM MgCl_2_*6H2O (hexahydrate) in ddH2O.

### Chromatin binding and whole cell staining studies (flow cytometry)

Cells were harvested with trypsin and spun at 1200 rpm for five minutes. Cells were then rinsed with PBS and spun down. For pre-extraction, cells were resuspended in 100 μL CSK buffer supplemented with 0.3% triton x-100 and HALT protease and phosphatase inhibitor cocktails, each at 1:100, for 10 minutes on ice (Thermo Scientific™ # 78442). For whole cell labeling, cells were set aside in PBS at RT. All cells were then spun down at 2000 g for 3 minutes and resuspended in 100% methanol for 15 minutes at RT. Cells were spun at 2000 g for 7 minutes and pre-extracted cells were set aside while whole cells were additionally permeabilized with 0.3% triton x-100 in PBS for five minutes at RT, then spun down. All cells were then resuspended in 100 μL immunofluorescence buffer (5% BSA + 0.5% NP-40 in PBS) with primary antibody: MCM-4 (Thermo Fisher Scientific Cat# MA5-52516, RRID:AB_3248993) (1:100), MCM-7 (Santa Cruz Biotechnology Cat# sc-9966, RRID:AB_627235) (1:25) for one hour at RT. Cells were then spun at 2000 g for 7 minutes and resuspended in 100 μL IF buffer containing secondary antibody (Abcam - Goat anti-mouse IgG H&L Alexa Fluor® 647 #ab150115 at 1:100; Invitrogen - Goat anti-Rabbit IgG (H + L) Highly Cross-Adsorbed Secondary Antibody, Alexa Fluor™ 647 #A31634A at 1:100) for 30 minutes protected from light. Cells were again spun, resuspended in PBS, and aliquoted to FACS tubes at which point RNAse A and DAPI were added and samples incubated for 45 minutes at RT before analyzing with an LSR Fortessa cytometer. 10,000 events were recorded per sample. For chromatin-loaded proteins, LSR Fortessa parameter/laser delays were set to DAPI for cell detection.

### Whole genome sequencing

Cells were seeded into 6 cm dishes with or without 2 μg/mL doxycycline for cyclin E overexpression and cultured for 72 h (to achieve ideal confluency), at which point TAK-931 was added for an additional 48 h. Cells were harvested with trypsin, rinsed once with PBS, then snap-frozen and stored at −80 °C. DNA was extracted from samples in accordance with the “MasterPure Complete DNA and RNA Purification Kit” from Biosearch Technologies (Cat# MC85200) and submitted for low depth 2×100 whole genomic sequencing.

### DNA replication timing profiling

Sequence data was aligned to human genome reference hg38 using BWA-MEM (Li,H. 2013. Aligning sequence reads, clone sequences and assembly contigs with BWAMEM. arXiv, 1303.3997). GC-corrected DNA copy number data were then generated with TIGER using a read length of 100 bp for alignability filtering and a bin size of 1000 bp. The median genome copy number was normalized to 2. The segment filtering module of TIGER was not implemented due to the high rate of copy number alterations in the cell lines. Instead, genomic windows with a copy number of less than 0.5 or more than 4 were removed. Subsequently, the copy number data was smoothed using default TIGER parameters to obtain DNA replication timing profiles [36]. Two independent biological replicates were performed.

### CRISPR screening

HCC-1806 cells were infected with the Toronto Knockout Version 3 (TKOv3) human CRISPR lentiviral library comprising 70,948 guides (4 guides/gene) targeting 18,053 protein-coding genes [37]. Cells were selected for successful incorporation of the sgRNA, and a small fraction was saved for whole genomic DNA for comparison of subsequent dropout rates to DMSO or drug-treated cells. Cells were passaged from P3-P8 in either 31.25 nM TAK-931 or 0.1% DMSO, at which point whole genomic DNA was harvested and submitted for sequencing. Raw fastq files were used as inputs for the Mageck pipeline (v0.5.9.5) for sgRNA counting and QC. The raw count file was then used as input for drugZ (v1.1.0.2) to calculate the sensitivity/resistance score to gene perturbation. The final output file contained gene rankings and associated statistics. A custom R script was used to produce a graphical representation of the drug screen results.

### Xenograft studies

All animal studies were conducted in compliance with the Institutional Animal Care and Use Committee (IACUC) at Roswell Park Comprehensive Cancer Center. HCC-1806 xenografts were developed by orthotopically injecting 1*10^6^ HCC-1806 cells into the mammary fat pads of female NOD scid gamma (NSG) mice aged 8 weeks. Tumor volume was measured using the formula: (MIN(Length:Width)^2^*MAX(Length:Width))/2. TAK-931 and BI-1347 were prepared in a vehicle (0.5% methycellulose) and thoroughly homogenized immediately prior to treatment. Drug preparations were maintained in the dark at RT or at 4 °C. Upon reaching an average tumor volume of 200–250 mm^3^, mice were randomly assigned into experimental and control cohorts, each of *n* = 4 mice. Oral gavage was used to deliver both TAK-931 and BI-1347 according to the indicated regimens: TAK-931 was delivered at 60 mg/kg once daily on a M/W/F regimen, and BI-1347 was delivered at 10 mg/kg once daily on an M-F regimen. Mice receiving combination treatment were administered with both TAK-931 and BI-1347 regimens. Vehicle mice received 0.5% methylcellulose once daily on a M-F regimen. Drug preparation was performed by the investigator, and oral gavage/measurements were performed by the lab technician blinded to the experimental hypothesis. Dosing regimens were determined using published literature, with TAK-931 administration modified to better capture synergy [29, 54, 66].

### Statistical analyses

All statistical analyses and determination of significance were conducted in GraphPad Prism v10 using the following thresholds: not significant (ns) *p* ≥ 0.05; *p < 0.05; **p < 0.01; ***p < 0.001; ****p < 0.0001. Individual statistical tests performed are listed in each corresponding figure legend. No inclusion/exclusion criteria were used for in vitro or in vivo studies. Power analyses were not conducted to determine sample sizes, as effect sizes were sufficient after assessing three replicates. All analyzed data were found to be normally distributed.

## Supplementary information

Supplementary Figures

## Data Availability

The data from the Broad Institute’s Cancer Dependency Map project that were analyzed in this study were obtained using the DepMap Public 25Q2 dataset and the listed genes and cell lines. Additionally, data analyzed from the Breast Invasive Carcinoma (TCGA, PanCancer Atlas) and Metabric were obtained from cBioPortal. The replication timing data generated in this study are available in the BioProject database under the following project ID: PRJNA1416898. All data presented in this study are available from the corresponding author upon request.
