# Supplementary material for: Cyclin E modulates vulnerability to CDC7 kinase inhibition
Source: Oncogenesis. 2026 Apr 24;15(1):27. doi: 10.1038/s41389-026-00613-5 (PMC13243581; doi:10.1038/s41389-026-00613-5)
Supplement: Supplementary file 1 — Supplementary Figures [file 41389_2026_613_MOESM1_ESM.pdf]

Figure S1

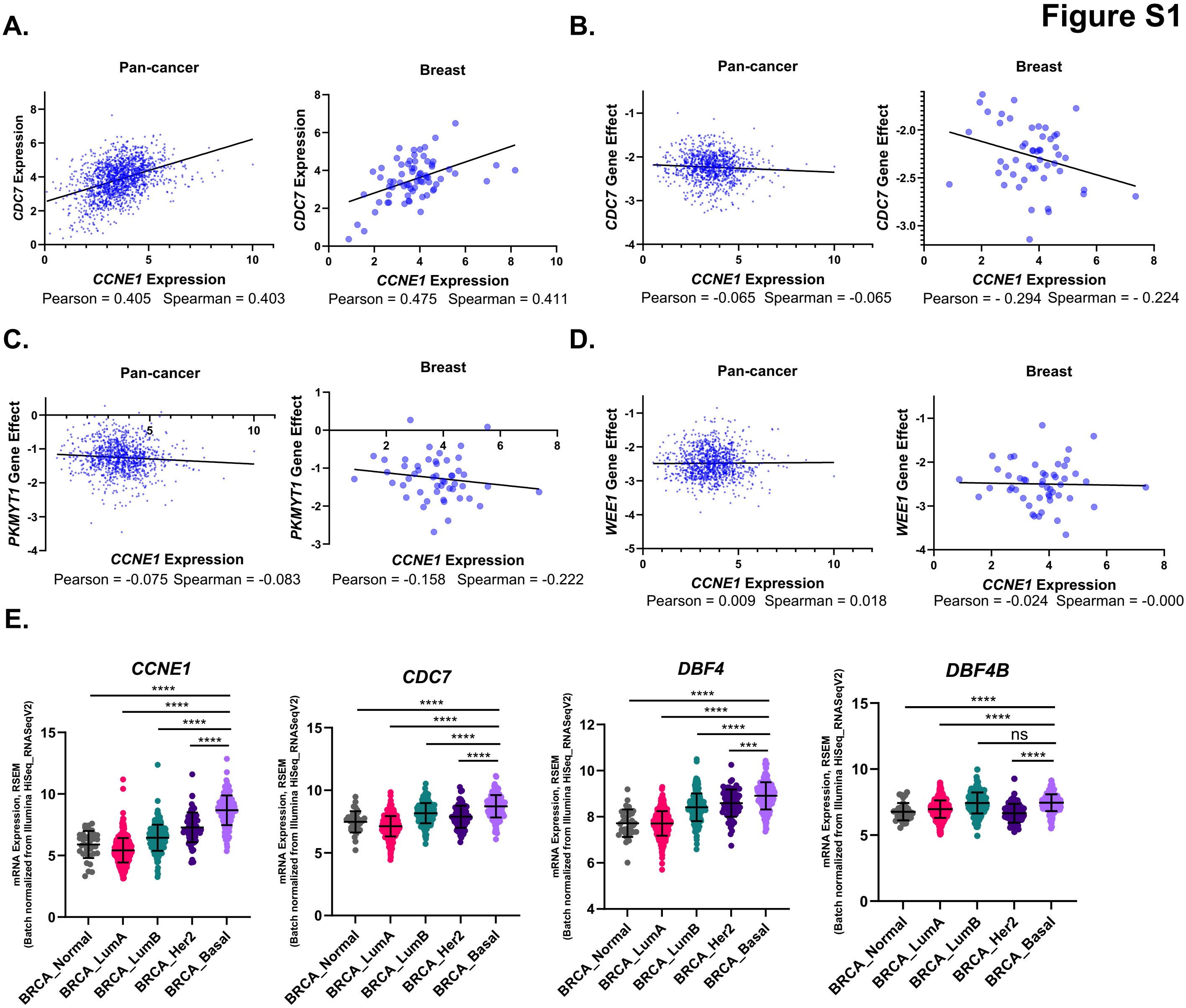
A.
Pan-cancer
CDC7 Expression
CCNE1 Expression
Pearson = 0.405 Spearman = 0.403
Breast
CDC7 Expression
CCNE1 Expression
Pearson = 0.475 Spearman = 0.411
B.
Pan-cancer
CDC7 Gene Effect
CCNE1 Expression
Pearson = -0.065 Spearman = -0.065
Breast
CDC7 Gene Effect
CCNE1 Expression
Pearson = - 0.294 Spearman = - 0.224
C.
Pan-cancer
PKMYT1 Gene Effect
CCNE1 Expression
Pearson = -0.075 Spearman = -0.083
Breast
PKMYT1 Gene Effect
CCNE1 Expression
Pearson = -0.158 Spearman = -0.222
D.
Pan-cancer
WEE1 Gene Effect
CCNE1 Expression
Pearson = 0.009 Spearman = 0.018
Breast
WEE1 Gene Effect
CCNE1 Expression
Pearson = -0.024 Spearman = -0.000
E.
CCNE1
CDC7
DBF4
DBF4B
mRNA Expression, RSEM (Batch normalized from Illumina HiSeq_RNASeqV2)
BRCA_Normal
BRCA_LumA
BRCA_LumB
BRCA_Her2
BRCA_Basal
****
***
ns

F.

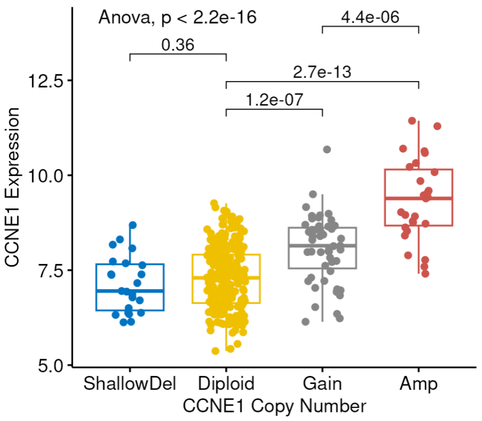
Anova, p < 2.2e-16
0.36
4.4e-06
2.7e-13
1.2e-07
CCNE1 Expression
12.5
10.0
7.5
5.0
ShallowDel
Diploid
Gain
Amp
CCNE1 Copy Number

G.

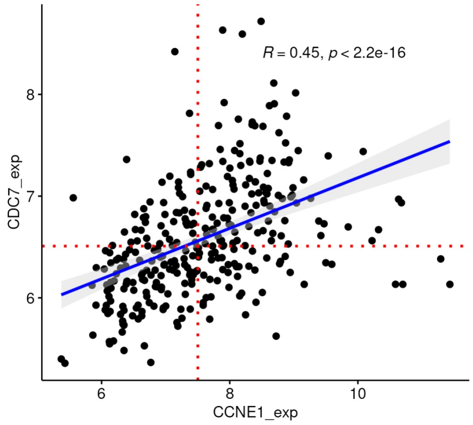
R = 0.45, p < 2.2e-16
CDC7_exp
8
7
6
6
8
10
CCNE1_exp

H.

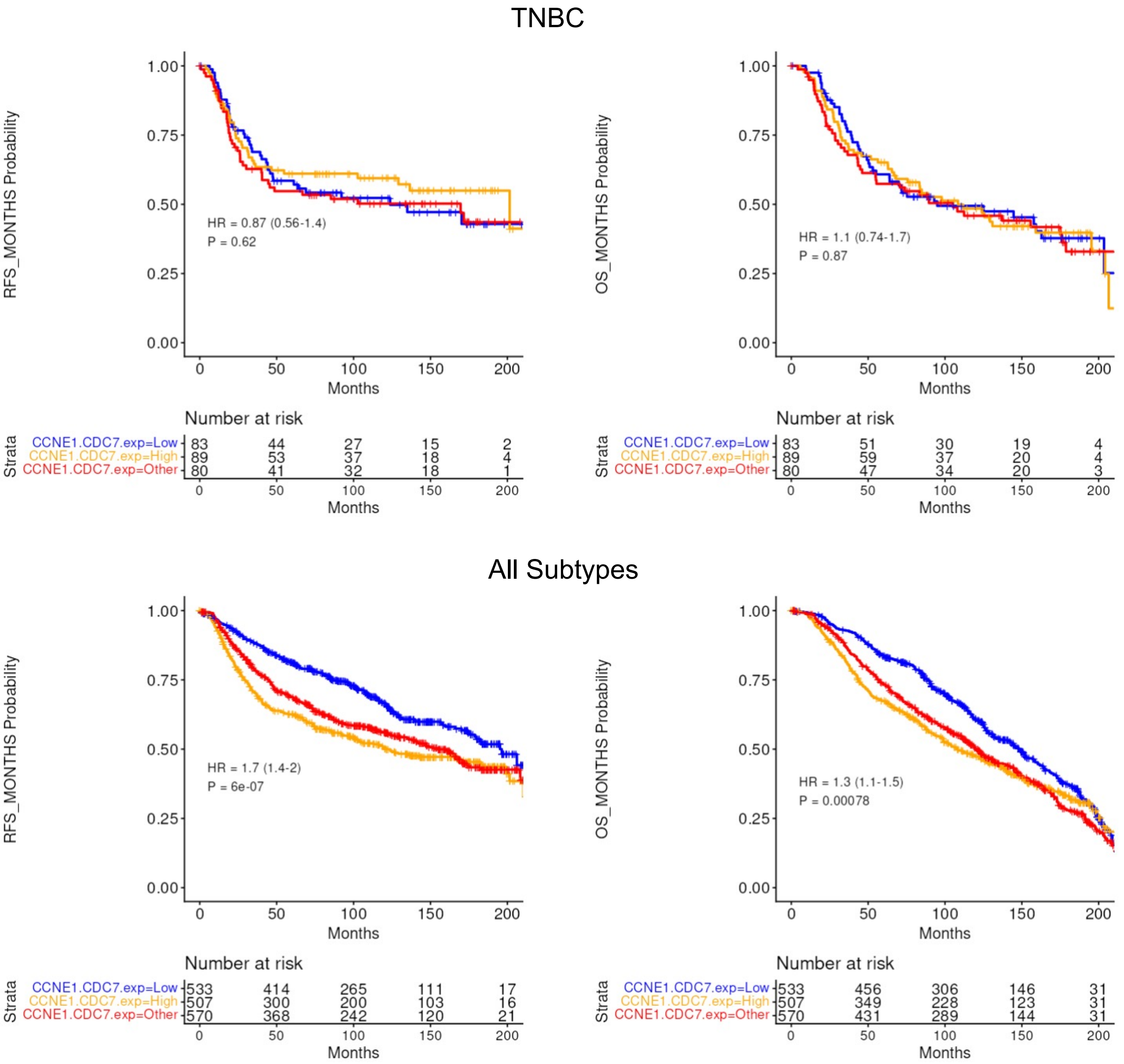
TNBC
RFS_MONTHS Probability
HR = 0.87 (0.56-1.4)
P = 0.62
Months
Number at risk
Strata
CCNE1.CDC7.exp=Low 83 44 27 15 2
CCNE1.CDC7.exp=High 89 53 37 18 4
CCNE1.CDC7.exp=Other 80 41 32 18 1
OS_MONTHS Probability
HR = 1.1 (0.74-1.7)
P = 0.87
CCNE1.CDC7.exp=Low 83 51 30 19 4
CCNE1.CDC7.exp=High 89 59 37 20 4
CCNE1.CDC7.exp=Other 80 47 34 20 3
All Subtypes
RFS_MONTHS Probability
HR = 1.7 (1.4-2)
P = 6e-07
CCNE1.CDC7.exp=Low 533 414 265 111 17
CCNE1.CDC7.exp=High 507 300 200 103 16
CCNE1.CDC7.exp=Other 570 368 242 120 21
OS_MONTHS Probability
HR = 1.3 (1.1-1.5)
P = 0.00078
CCNE1.CDC7.exp=Low 533 456 306 146 31
CCNE1.CDC7.exp=High 507 349 228 123 31
CCNE1.CDC7.exp=Other 570 431 289 144 31

Figure S1

I.

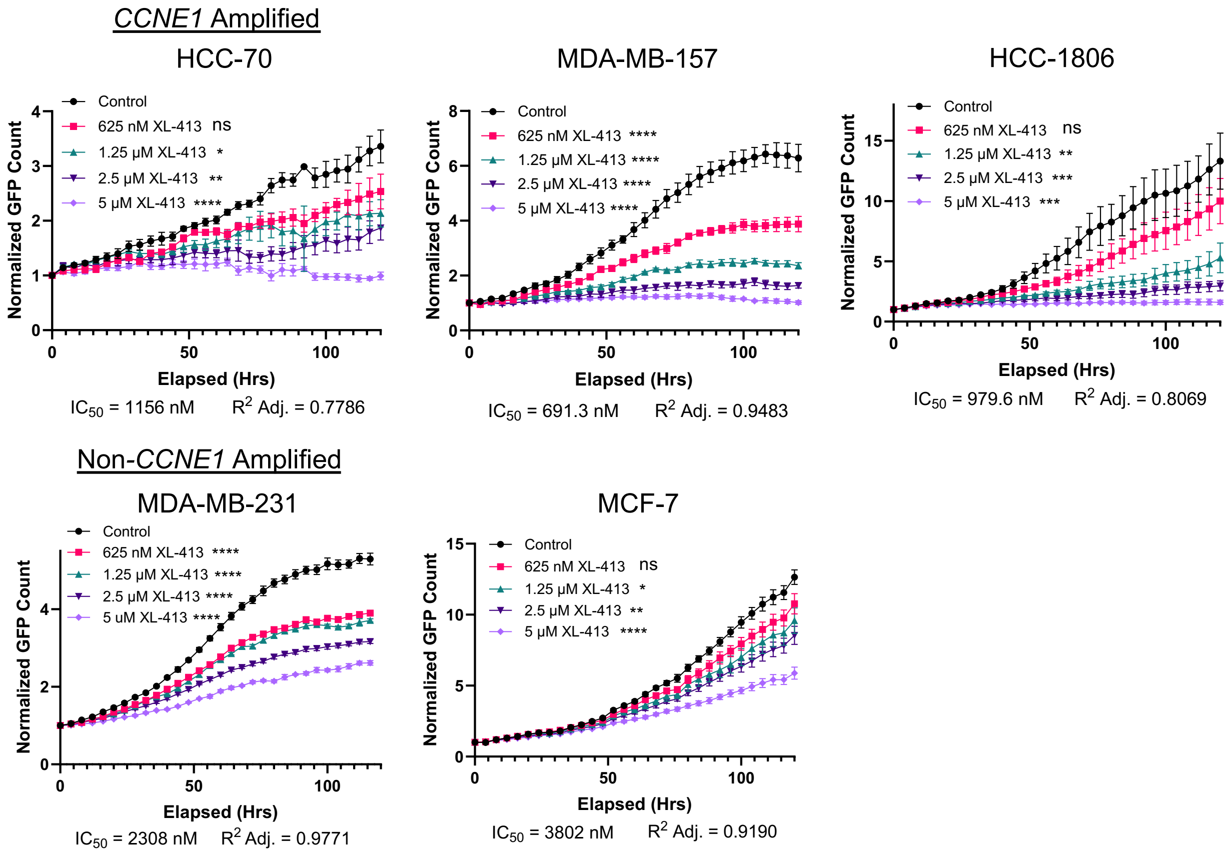
CCNE1 Amplified
HCC-70
Control
625 nM XL-413 ns
1.25 μM XL-413 *
2.5 μM XL-413 **
5 μM XL-413 ****
Normalized GFP Count
Elapsed (Hrs)
IC50 = 1156 nM R2 Adj. = 0.7786
MDA-MB-157
Control
625 nM XL-413 ****
1.25 μM XL-413 ****
2.5 μM XL-413 ****
5 μM XL-413 ****
Normalized GFP Count
Elapsed (Hrs)
IC50 = 691.3 nM R2 Adj. = 0.9483
HCC-1806
Control
625 nM XL-413 ns
1.25 μM XL-413 **
2.5 μM XL-413 ***
5 μM XL-413 ***
Normalized GFP Count
Elapsed (Hrs)
IC50 = 979.6 nM R2 Adj. = 0.8069
Non-CCNE1 Amplified
MDA-MB-231
Control
625 nM XL-413 ****
1.25 μM XL-413 ****
2.5 μM XL-413 ****
5 uM XL-413 ****
Normalized GFP Count
Elapsed (Hrs)
IC50 = 2308 nM R2 Adj. = 0.9771
MCF-7
Control
625 nM XL-413 ns
1.25 μM XL-413 *
2.5 μM XL-413 **
5 μM XL-413 ****
Normalized GFP Count
Elapsed (Hrs)
IC50 = 3802 nM R2 Adj. = 0.9190

J.

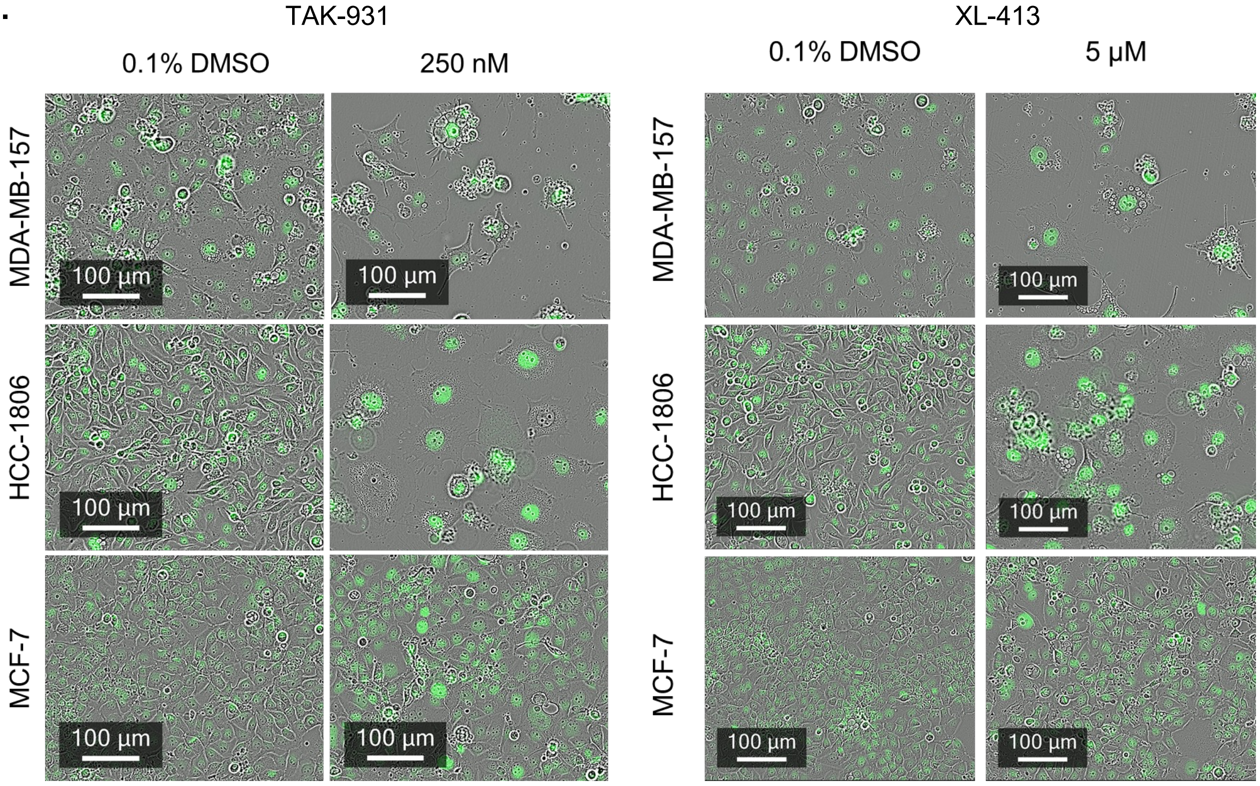
TAK-931
0.1% DMSO
250 nM
XL-413
0.1% DMSO
5 μM
MDA-MB-157
HCC-1806
MCF-7
100 μm

Figure S1

K.

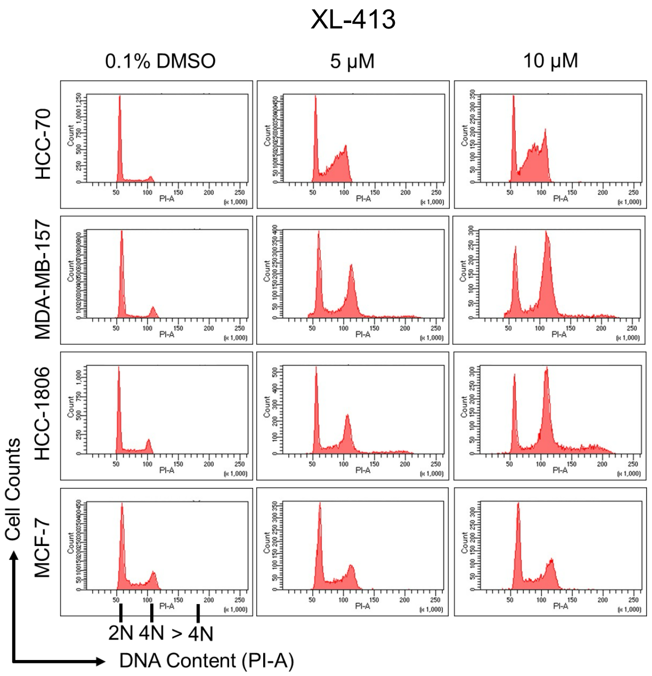

L.

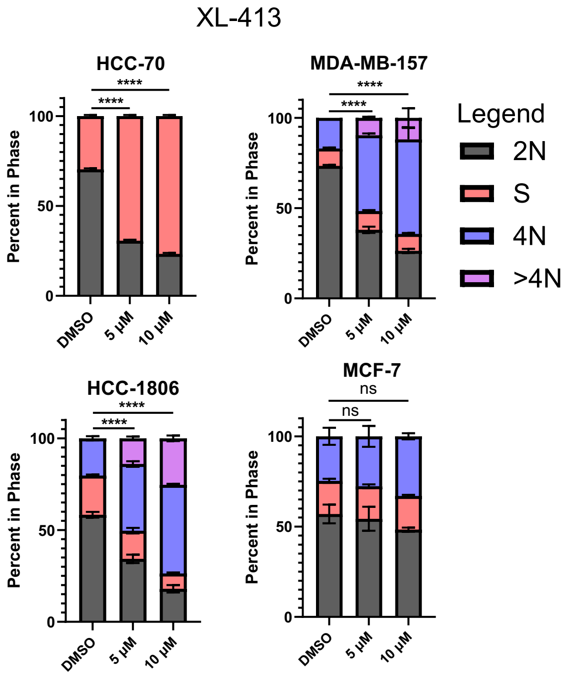

M.

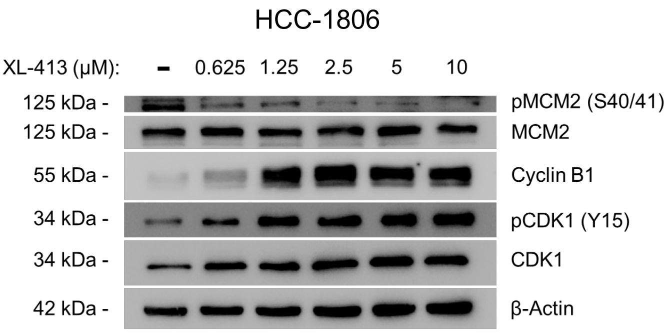

N.

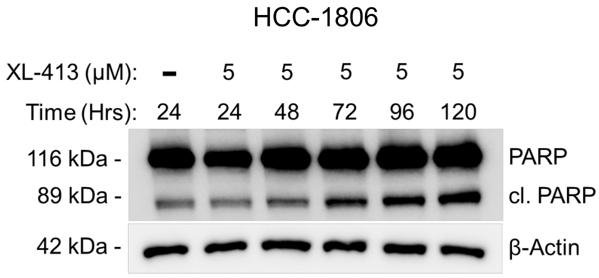

O.

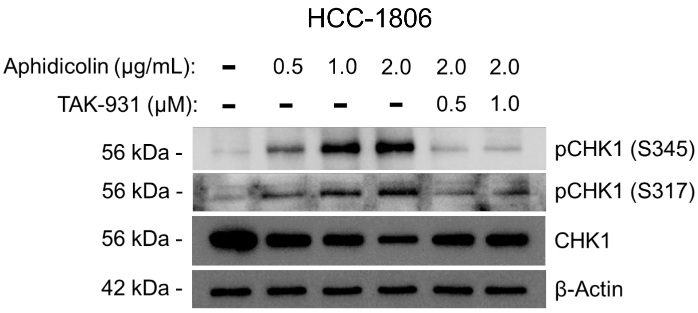

Supplemental Figure legends

Figure S1. CDC7 Inhibition is Efficacious in *CCNE1* Amplified TNBC.

A, Line graph derived from the DepMap project for *CDC7* expression as a function of *CCNE1* expression in the pan-cancer (left) or breast cancer (right) settings. **B,** Line graph derived from the DepMap project for *CDC7* gene effect as a function of *CCNE1* expression in the pan-cancer (left) or breast cancer (right) settings. **C,** Line graph derived from the DepMap project for *PKMYT1* gene effect as a function of *CCNE1* expression in the pan-cancer (left) or breast cancer (right) settings. **D,** Line graph derived from the DepMap project for *WEE1* gene effect as a function of *CCNE1* expression in the pan-cancer (left) or breast cancer (right) settings. **E,** Bar graphs derived from the cancer genome atlas for *CCNE1*, *CDC7*, *DBF4*, and *DBF4B* transcript levels across different breast cancer subtypes (BRCA_Normal, n=36; BRCA_LumA, n=499; BRCA_LumB, n=197; BRCA_Her2, n=78; BRCA_Basal, n=171; one-way ANOVA with Tukey's multiple comparisons test; lines indicate mean with SD). **F,** *CCNE1* status and associated expression in TNBC patients from Metabric. **G,** Correlation of *CCNE1* and *CDC7* expression in TNBC patients from Metabric. **H,** Kaplan-Meier plots of recurrence-free survival (RFS, left) and overall survival (OS, right) of TNBC (top) and all breast cancer patients (bottom) dichotomized by median *CCNE1* and *CDC7* expression. HR, hazard ratio. **I,** *CCNE1* amplified or non-amplified breast cancer cells treated with serial concentrations of XL-413 for five days and monitored with Cellcyte or Incucyte live cell proliferative software (n=4; one-way ANOVA with Dunnett's multiple comparisons test comparing each concentration to DMSO control using endpoint normalized GFP counts; error bars represent SEM). **J,** Representative images captured at the end of five-day live cell proliferative analyses from cells treated with 250 nM TAK-931 (left) or 5 μM XL-413 (right). **K,** Representative cell cycle profiling of the indicated cell lines following 48 hr treatment with XL-413. **L,** Quantification of cell cycle state from indicated cell lines treated with 48 hr XL-413 (n=3; one-way ANOVA with Dunnett's multiple comparisons

test comparing each concentration to DMSO control with significance determined for ≥ 4N DNA (for HCC-70, significance determined for >2N DNA); error bars represent SD). **M,** Representative western blot of HCC-1806 cells for pMCM2 (S40/41) and mitotic inhibition following 48 hr XL-413 treatment. **N,** Representative western blot of HCC-1806 cells for cleaved PARP following time-dependent exposure to 5 μM XL-413. **O,** Representative western blot of HCC-1806 cells for pCHK1 (S317, S345) following 24 hr aphidicolin treatment with or without TAK-931.

Figure S2

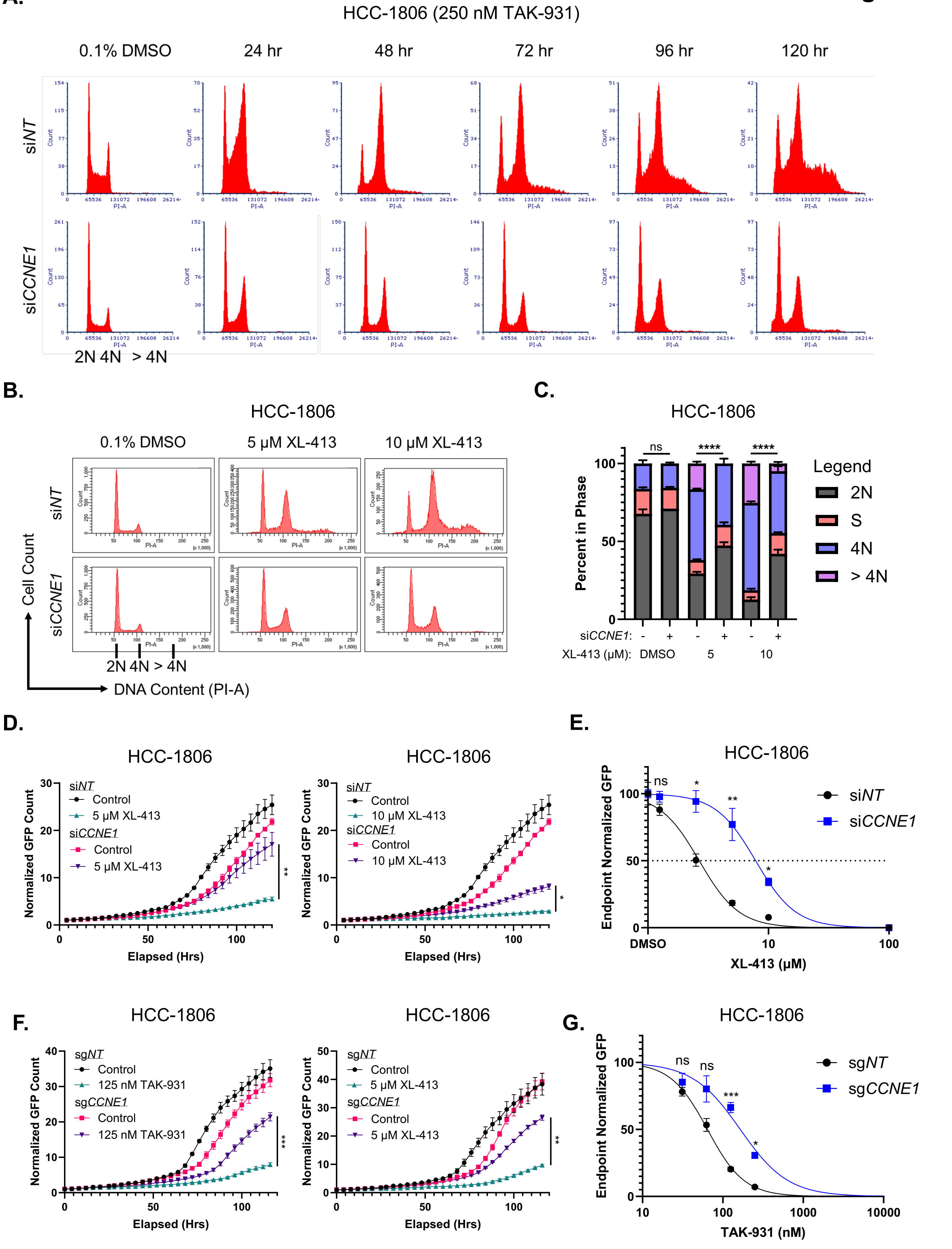
A.
HCC-1806 (250 nM TAK-931)
0.1% DMSO
24 hr
48 hr
72 hr
96 hr
120 hr
siNT
siCCNE1
Count
PI-A
2N 4N > 4N
B.
HCC-1806
0.1% DMSO
5 μM XL-413
10 μM XL-413
siNT
siCCNE1
Cell Count
DNA Content (PI-A)
2N 4N > 4N
C.
HCC-1806
Percent in Phase
ns
****
****
Legend
2N
S
4N
> 4N
siCCNE1: - + - + - +
XL-413 (μM): DMSO 5 10
D.
HCC-1806
Normalized GFP Count
Elapsed (Hrs)
siNT
Control
5 μM XL-413
siCCNE1
Control
5 μM XL-413
**
HCC-1806
siNT
Control
10 μM XL-413
siCCNE1
Control
10 μM XL-413
*
E.
HCC-1806
Endpoint Normalized GFP
XL-413 (μM)
DMSO
10
100
ns
*
**
*
siNT
siCCNE1
F.
HCC-1806
Normalized GFP Count
Elapsed (Hrs)
sgNT
Control
125 nM TAK-931
sgCCNE1
Control
125 nM TAK-931
***
HCC-1806
sgNT
Control
5 μM XL-413
sgCCNE1
Control
5 μM XL-413
**
G.
HCC-1806
Endpoint Normalized GFP
TAK-931 (nM)
ns
ns
***
*
sgNT
sgCCNE1

Figure S2

H.

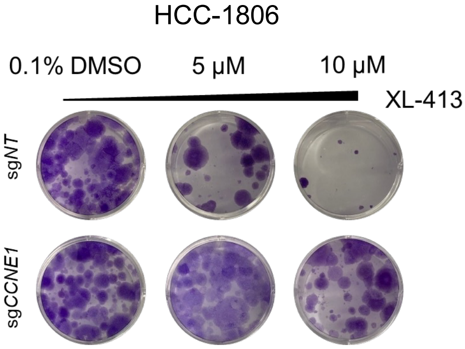
HCC-1806
0.1% DMSO
5 μM
10 μM
XL-413
sgNT
sgCCNE1

I.

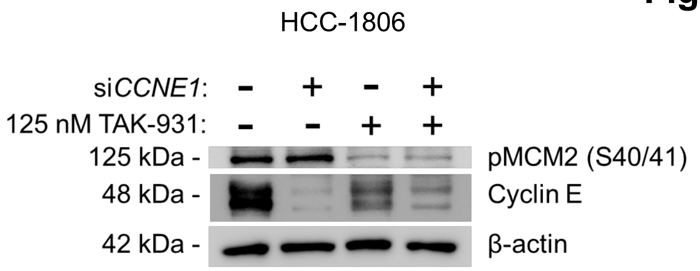
HCC-1806
siCCNE1:
125 nM TAK-931:
125 kDa -
48 kDa -
42 kDa -
pMCM2 (S40/41)
Cyclin E
β-actin

Figure S2. *CCNE1* Knockdown Reverses Sensitivity to CDC7 Inhibition.

A, Representative cell cycle profiling of HCC-1806 cells following *CCNE1* transcriptional knockdown and time-dependent exposure to 250 nM TAK-931. **B,** Representative cell cycle profiling of HCC-1806 cells following *CCNE1* transcriptional knockdown and 48 hr treatment with indicated concentrations of XL-413. **C,** Quantification of cell cycle state from HCC-1806 cells following *CCNE1* transcriptional knockdown and 48 hr treatment with indicated concentrations of XL-413 (n=3; two-way ANOVA with Tukey's multiple comparisons test accounting for gene and drug effect with significance determined for ≥ 4N DNA; error bars represent SD). **D,**HCC-1806 cells following *CCNE1* knockdown and treatment with 5 μM (left) or 10 μM (right) XL-413 for five days and monitored with Cellcyte or Incucyte live cell proliferative software (n=4; two-way ANOVA with Tukey's multiple comparisons test accounting for gene and drug effect using endpoint normalized GFP counts; error bars represent SEM). **E,** IC_{50} curves derived from live cell proliferative analyses as described in panel **D** (n=4; two-way ANOVA with Tukey's multiple comparisons test accounting for gene and drug effect using endpoint normalized GFP counts; error bars represent SEM)**F,** HCC-1806 CRISPR *CCNE1* isogenic pair following treatment with 125 nM TAK-931 (left) or 5 μM XL-413 (right) for five days and monitored with Cellcyte or Incucyte live cell proliferative software (n=4; two-way ANOVA with Tukey's multiple comparisons test accounting for gene and drug effect using endpoint normalized GFP counts; error bars represent SEM) . **G,** IC_{50} curves derived from TAK-931 live cell proliferative analysis as described in panel **F**(n=4; two-way ANOVA with Tukey's multiple comparisons test accounting for gene and drug effect using endpoint normalized GFP counts; error bars represent SEM) . **H,** Representative colony outgrowth assay from HCC-1806 cells with CRISPR deleted *CCNE1* or non-targeting control and treatment with indicated concentrations of XL-413 for three cycles, with treatment replenishment every five days. **I,** Representative western blot of HCC-1806 cells for pMCM2 (S40/41) and cyclin E1 following *CCNE1* transcriptional knockdown and 24 hr treatment with 125 nM TAK-931.

Figure S3

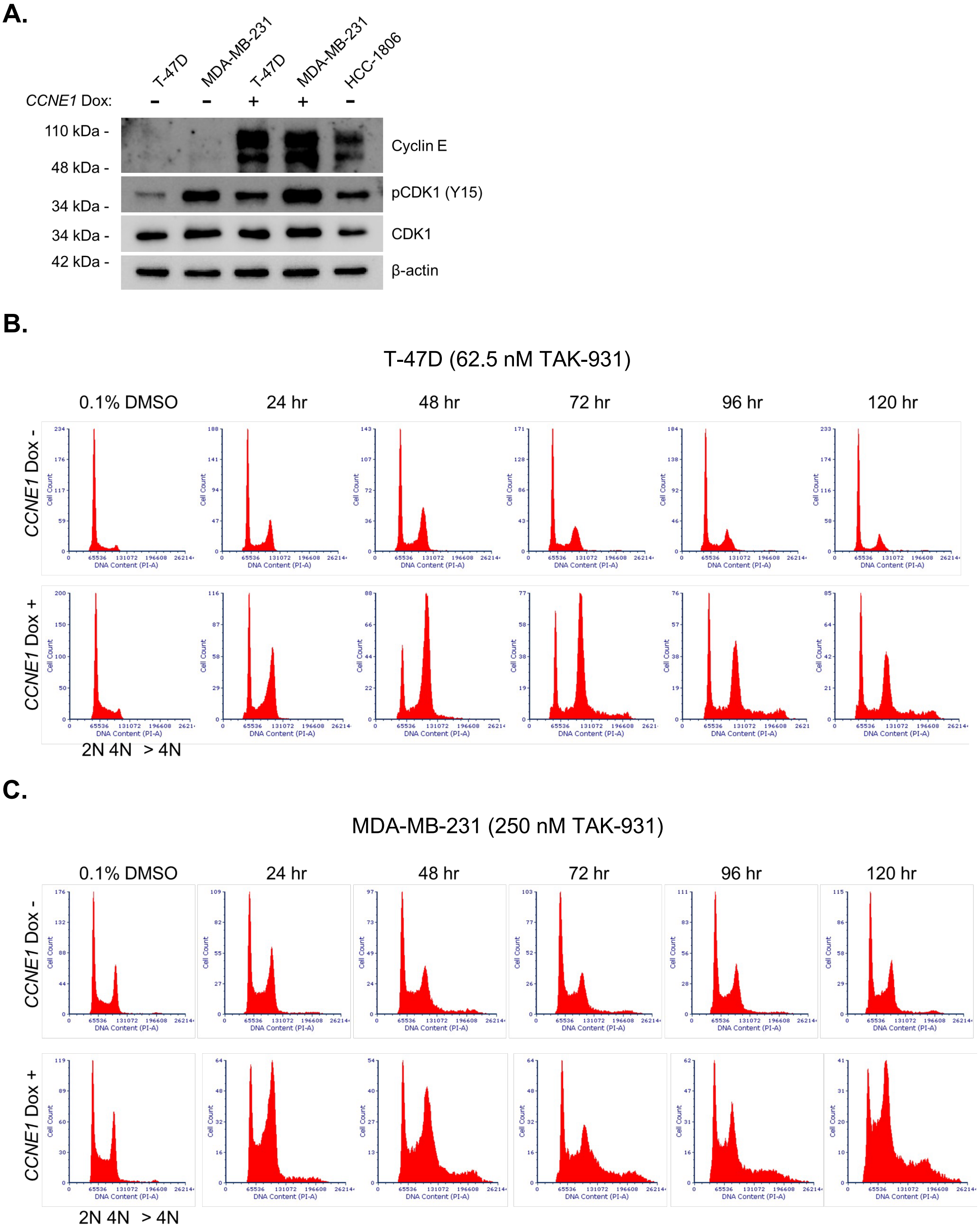
A.
T-47D
MDA-MB-231
T-47D
MDA-MB-231
HCC-1806
CCNE1 Dox:
–
–
+
+
–
110 kDa -
48 kDa -
Cyclin E
34 kDa -
pCDK1 (Y15)
34 kDa -
CDK1
42 kDa -
β-actin
B.
T-47D (62.5 nM TAK-931)
0.1% DMSO
24 hr
48 hr
72 hr
96 hr
120 hr
CCNE1 Dox -
CCNE1 Dox +
Cell Count
DNA Content (PI-A)
2N 4N > 4N
C.
MDA-MB-231 (250 nM TAK-931)
0.1% DMSO
24 hr
48 hr
72 hr
96 hr
120 hr
CCNE1 Dox -
CCNE1 Dox +
Cell Count
DNA Content (PI-A)
2N 4N > 4N

Figure S3

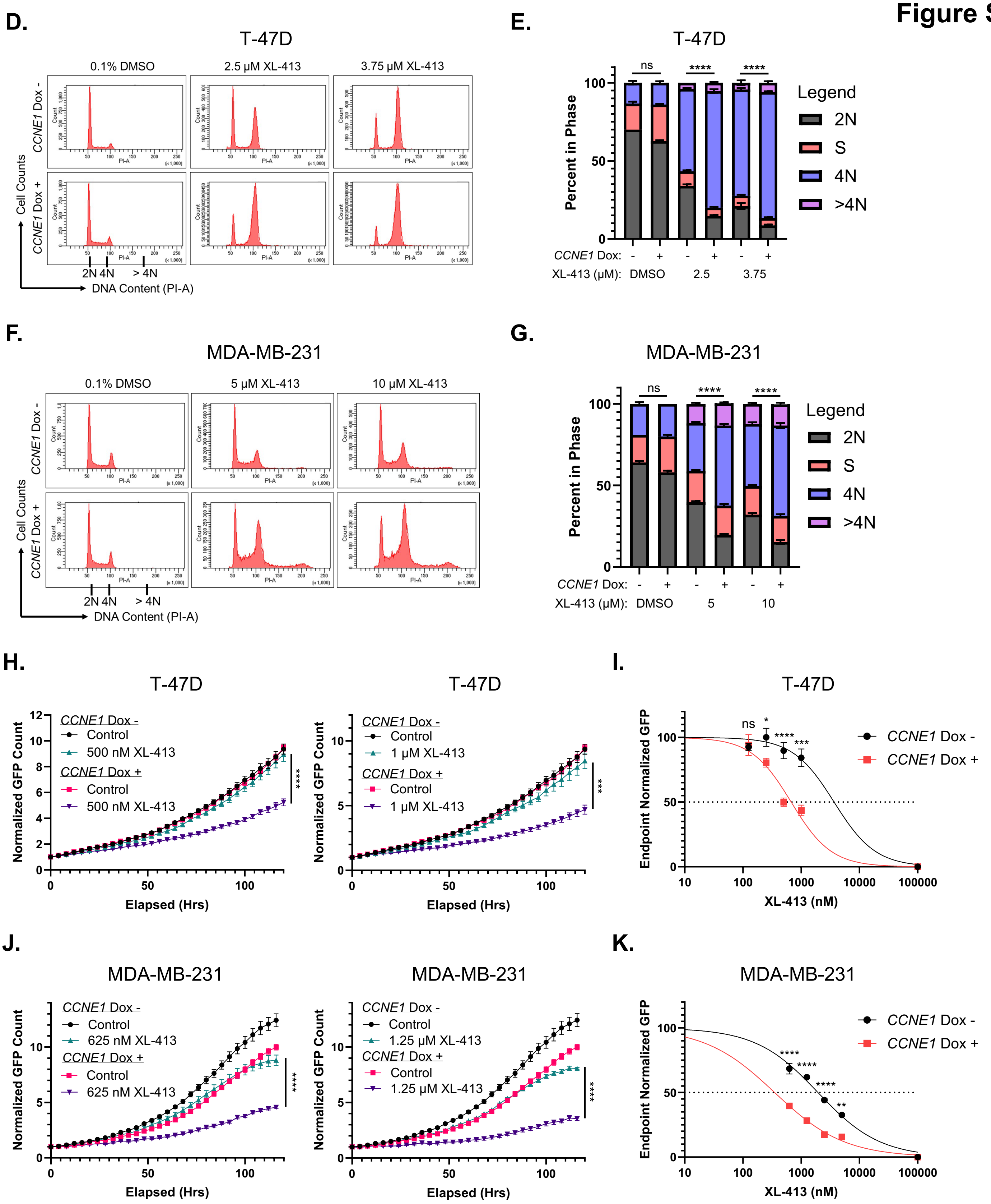

D.
T-47D
0.1% DMSO
2.5 μM XL-413
3.75 μM XL-413
CCNE1 Dox -
CCNE1 Dox +
Cell Counts
2N 4N
> 4N
DNA Content (PI-A)
E.
T-47D
Percent in Phase
ns
****
****
Legend
2N
S
4N
>4N
CCNE1 Dox:
XL-413 (μM):
DMSO
2.5
3.75
F.
MDA-MB-231
0.1% DMSO
5 μM XL-413
10 μM XL-413
G.
MDA-MB-231
5
10
H.
T-47D
Normalized GFP Count
CCNE1 Dox -
Control
500 nM XL-413
CCNE1 Dox +
1 μM XL-413
Elapsed (Hrs)
I.
Endpoint Normalized GFP
XL-413 (nM)
J.
625 nM XL-413
1.25 μM XL-413
K.

Figure S3

L.

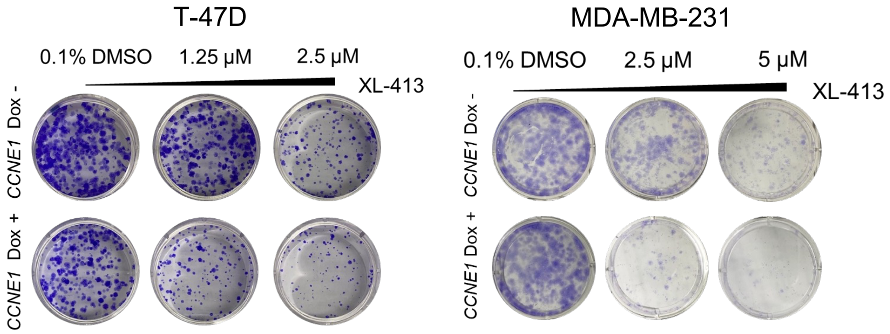

M.

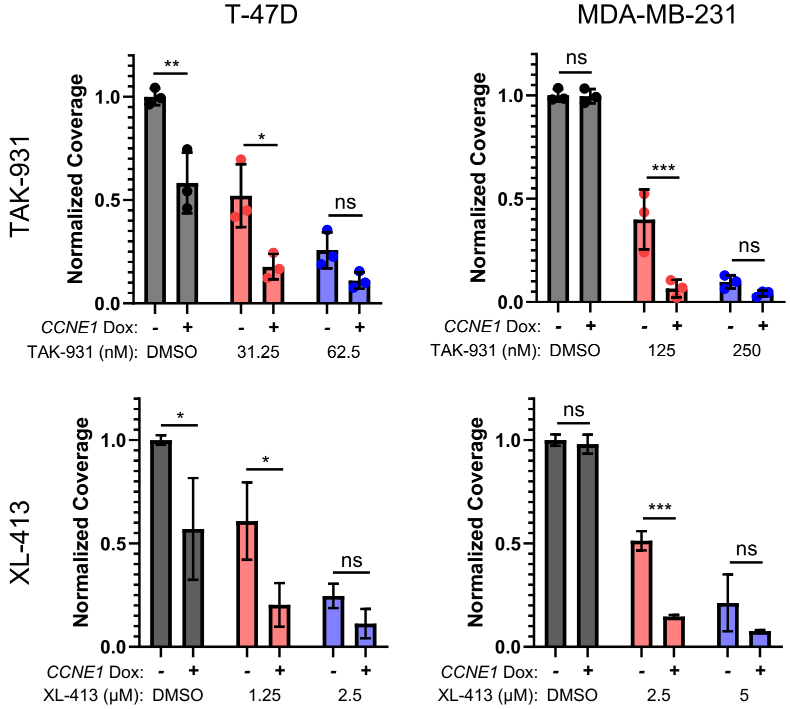

N.

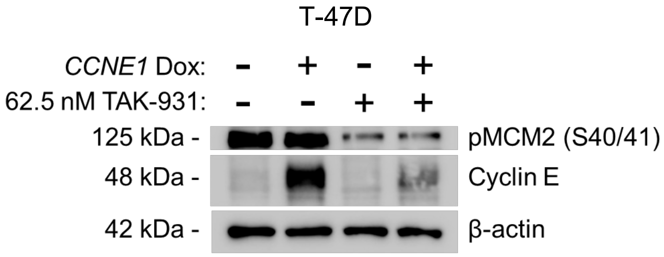

Figure S3. Cyclin E Overexpression Enhances Sensitivity to CDC7 Inhibition.

A, Representative western blot of indicated cell lines for pCDK1 (Y15) and cyclin E1 following 24 hr induction of *CCNE1*. **B,** Representative cell cycle profiling of T-47D cells following cyclin E overexpression and time-dependent exposure to 62.5 nM TAK-931. **C,** Representative cell cycle profiling of MDA-MB-231 cells following cyclin E overexpression and time-dependent exposure to 250 nM TAK-931. **D,** Representative cell cycle profiling of T-47D cells following cyclin E overexpression and 48 hr treatment with indicated concentrations of XL-413. **E,** Quantification of cell cycle state from T-47D cells following cyclin E overexpression and 48 hr treatment with indicated concentrations of XL-413 (n=3; two-way ANOVA with Tukey's multiple comparisons test accounting for gene and drug effect with significance determined for ≥ 4N DNA; error bars represent SD). **F,** Representative cell cycle profiling of MDA-MB-231 cells following cyclin E overexpression and 48 hr treatment with indicated concentrations of XL-413 . **G,** Quantification of cell cycle state from MDA-MB-231 cells following cyclin E overexpression and 48 hr treatment with indicated concentrations of XL-413 (n=3; two-way ANOVA with Tukey's multiple comparisons test accounting for gene and drug effect with significance determined for ≥ 4N DNA; error bars represent SD). **H,** Representative T-47D cells following cyclin E overexpression and treatment with 500 nM (left) or 1 μM (right) XL-413 for five days and monitored with Cellcyte or Incucyte live cell proliferative software (n=4; two-way ANOVA with Tukey's multiple comparisons test accounting for gene and drug effect using endpoint normalized GFP counts; error bars represent SEM). **I,** IC_{50} curves derived from live cell proliferative analyses as described in panel **H** (n=4; two-way ANOVA with Tukey's multiple comparisons test accounting for gene and drug effect using endpoint normalized GFP counts; error bars represent SEM) . **J,** Representative MDA-MB-231 cells following cyclin E overexpression and treatment with 625 nM (left) or 1.25 μM (right) XL-413 for five days and monitored with Cellcyte or Incucyte live cell proliferative software (n=4; two-way ANOVA with Tukey's multiple comparisons test accounting for gene and drug effect using endpoint normalized GFP counts; error bars represent SEM) . **K,**

IC_{50} curves derived from live cell proliferative analyses as described in panel **J** (n=4; two-way ANOVA with Tukey's multiple comparisons test accounting for gene and drug effect using endpoint normalized GFP counts; error bars represent SEM) . **L,** Representative colony outgrowth assays from T-47D (left) and MDA-MB-231 (right) cells following cyclin E overexpression and treatment with indicated concentrations of XL-413 for three cycles, with doxycycline and treatment replenishment every five days. **M,** Normalized well coverage by cell colonies from TAK-931 and XL-413 colony outgrowth assays in T-47D and MDA-MB-231 cells (n=3; two-way ANOVA with Tukey's multiple comparisons test accounting for gene and drug effect using endpoint normalized well coverage; error bars represent SD). **N,** Representative western blot of T-47D cells for pMCM2 (S40/41) and cyclin E1 following cyclin E overexpression and 24 hr treatment with 62.5 nM TAK-931.

Figure S4

A.

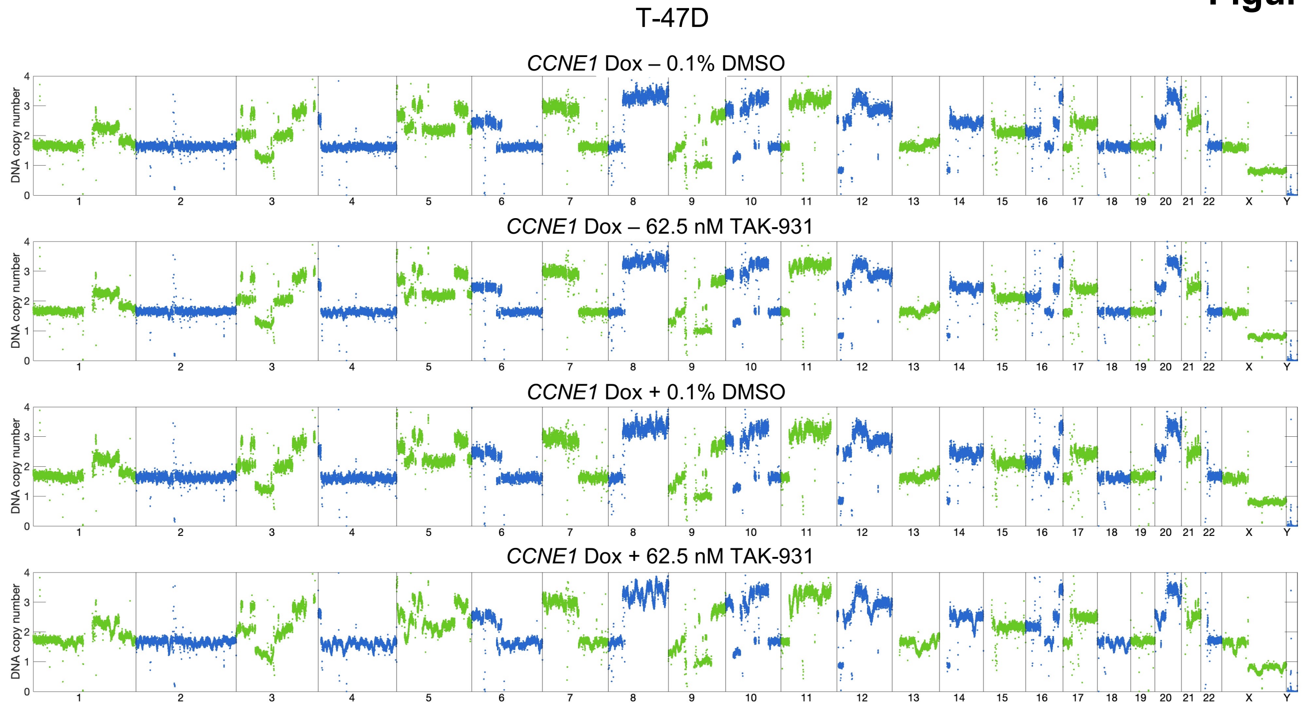
T-47D
CCNE1 Dox – 0.1% DMSO
CCNE1 Dox – 62.5 nM TAK-931
CCNE1 Dox + 0.1% DMSO
CCNE1 Dox + 62.5 nM TAK-931
DNA copy number
1 2 3 4 5 6 7 8 9 10 11 12 13 14 15 16 17 18 19 20 21 22 X Y

B.

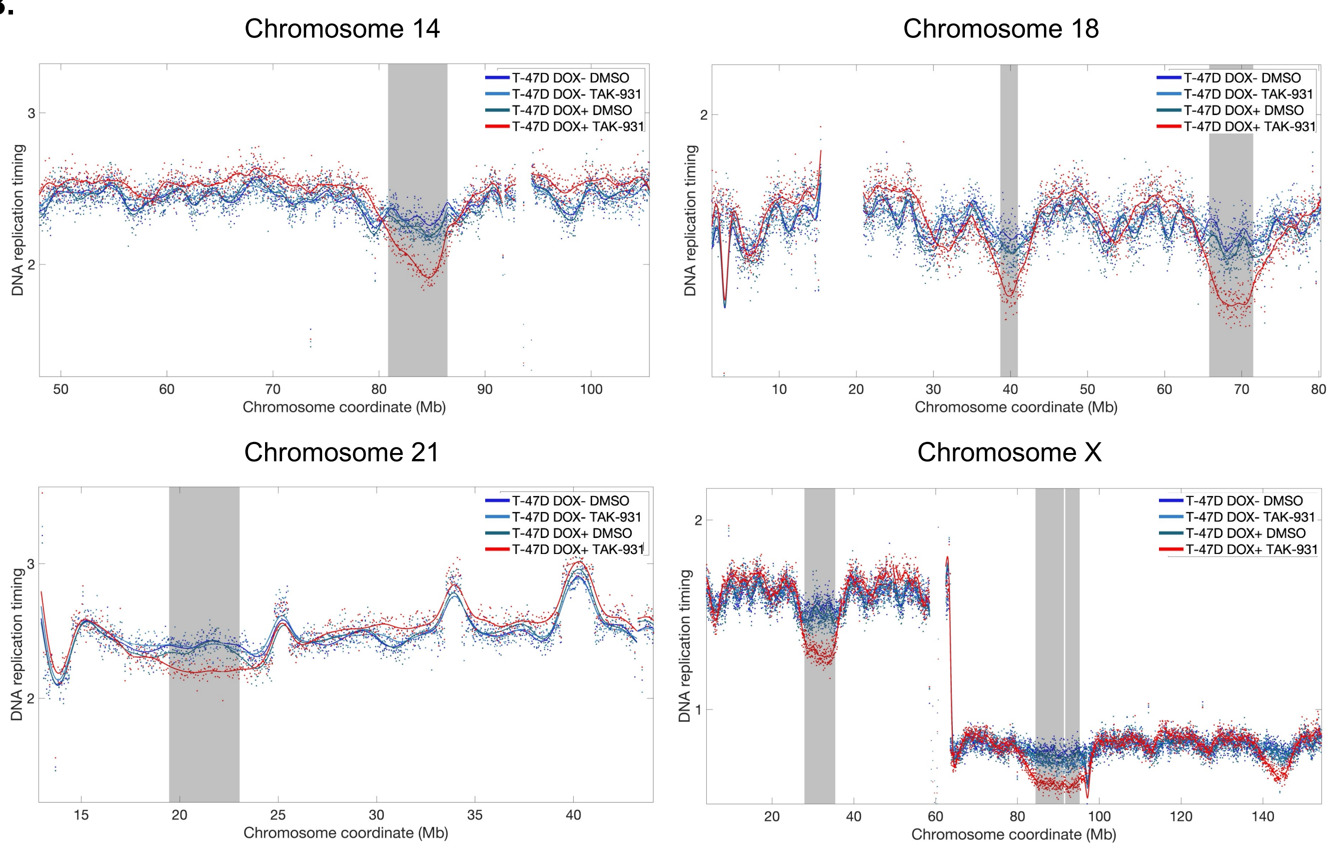
Chromosome 14
Chromosome 18
Chromosome 21
Chromosome X
T-47D DOX- DMSO
T-47D DOX- TAK-931
T-47D DOX+ DMSO
T-47D DOX+ TAK-931
DNA replication timing
Chromosome coordinate (Mb)

Figure S4

C.

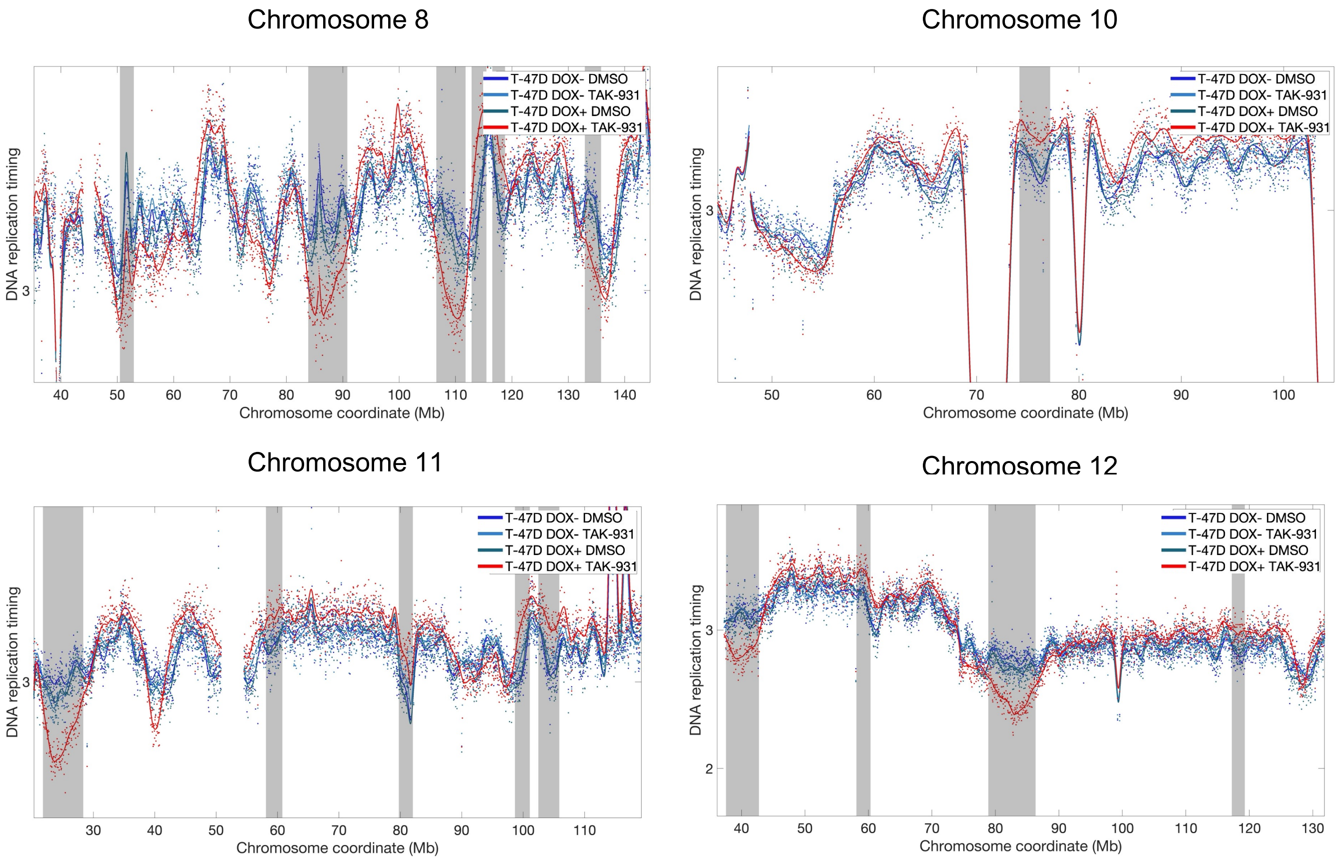

Chromosome 8
Chromosome 10
Chromosome 11
Chromosome 12
T-47D DOX- DMSO
T-47D DOX- TAK-931
T-47D DOX+ DMSO
T-47D DOX+ TAK-931
DNA replication timing
Chromosome coordinate (Mb)

Figure S4. CDC7 Inhibition Associates with Delayed Replication Timing in Cyclin E Deregulated Cells.

A, Representative individual genome-wide replication timing profiles from T-47D cells following cyclin E overexpression and 48 hr treatment with 62.5 nM TAK-931. **B,** Representative individual chromosome replication timing profiles from T-47D cells following cyclin E overexpression and 48 hr treatment with 62.5 nM TAK-931. **C,** Representative individual chromosome replication timing profiles from T-47D cells following cyclin E overexpression and 48 hr treatment with 62.5 nM TAK-931.

Figure S5

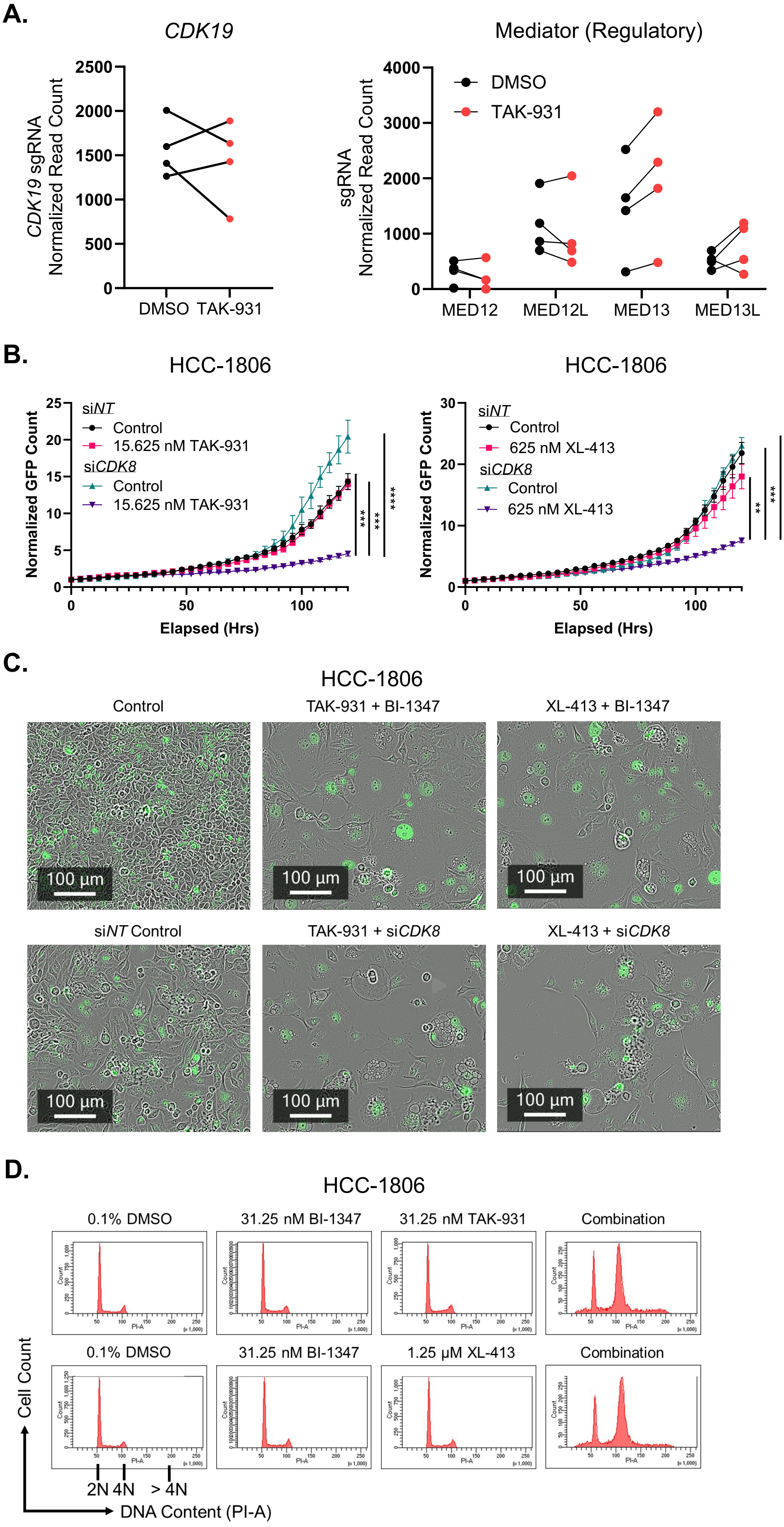
A.
CDK19
Mediator (Regulatory)
CDK19 sgRNA Normalized Read Count
sgRNA Normalized Read Count
2500
2000
1500
1000
500
0
4000
3000
2000
1000
0
DMSO
TAK-931
MED12
MED12L
MED13
MED13L
B.
HCC-1806
HCC-1806
Normalized GFP Count
Elapsed (Hrs)
siNT
Control
15.625 nM TAK-931
siCDK8
Control
15.625 nM TAK-931
625 nM XL-413
Control
625 nM XL-413
25
20
15
10
5
0
30
50
100
***
***
****
**
***
****
C.
HCC-1806
Control
TAK-931 + BI-1347
XL-413 + BI-1347
siNT Control
TAK-931 + siCDK8
XL-413 + siCDK8
100 µm
D.
HCC-1806
0.1% DMSO
31.25 nM BI-1347
31.25 nM TAK-931
Combination
0.1% DMSO
31.25 nM BI-1347
1.25 µM XL-413
Combination
Cell Count
2N 4N
> 4N
DNA Content (PI-A)

Figure S5

E.

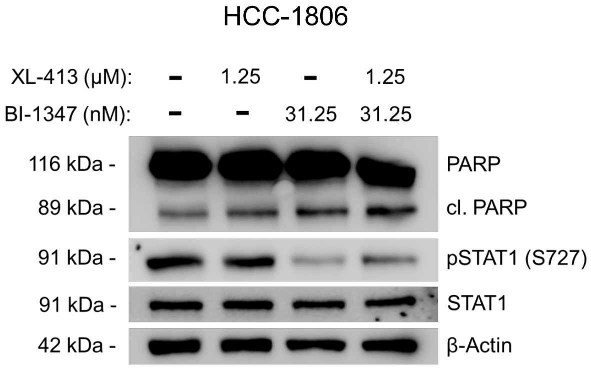

F.

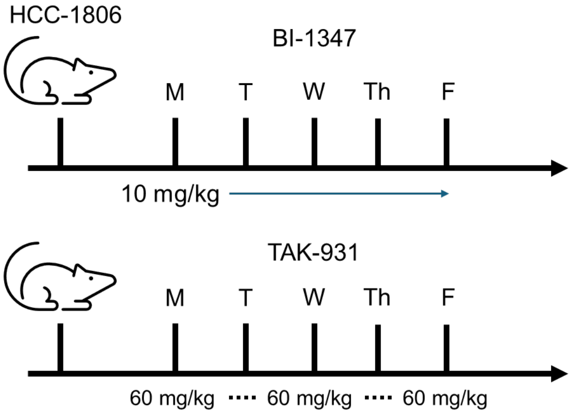

G.

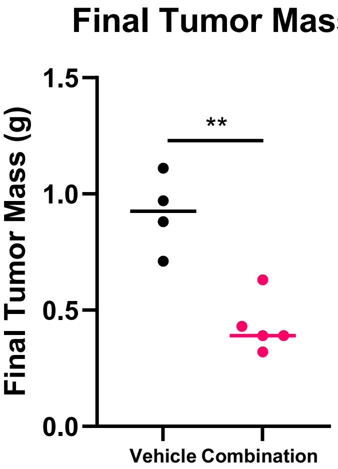

Figure S5. CDK8 Inhibition Synergizes with CDC7 Inhibition in *CCNE1*-amplified Cells.

A, Individual guide dropout rates for *CDK19* (left)and regulatory mediator complex members (right) from HCC-1806 cells following CRISPR screening. **B,** Representative HCC-1806 cells following *CDK8* knockdown and treatment with 15.625 nM TAK-931 (left) or 625 nM XL-413 (right) for five days and monitored with Cellcyte or Incucyte live cell proliferative software (n=4; one-way ANOVA with Tukey's multiple comparisons test comparing each treatment condition using endpoint normalized GFP counts; error bars represent SEM). **C,** Representative images captured at the end of five-day live cell proliferative analyses from HCC-1806 cells following treatment with BI-1347 and TAK-931 or XL-413 (top) or *CDK8* knockdown and treatment with TAK-931 or XL-413 (bottom). **D,** Representative cell cycle profiling of HCC-1806 cells following 48 hr treatment with BI-1347, TAK-931 or combination (top) or BI-1347, XL-413 or combination (bottom). **E,** Representative western blot of HCC-1806 cells for cleaved PARP and pSTAT1 (S727) following treatment with combined XL-413 and BI-1347 for 120 hrs. **F,** Schematic depicting treatment regimen for HCC-1806 cell line derived xenograft study for *in vivo* efficacy of combined TAK-931 and BI-1347. **G,** Final tumor mass from *in vivo* HCC-1806 xenograft models orally administered combined TAK-931 and BI-1347 (n=4 per vehicle group and 5 per combination group; unpaired t-test with Welch's correction (two-tailed)).
